# Transcriptome Profiling of *Staphylococcus aureus* Associated Extracellular Vesicles Reveals Presence of Small RNA-Cargo

**DOI:** 10.3389/fmolb.2020.566207

**Published:** 2021-01-13

**Authors:** Bishnu Joshi, Bhupender Singh, Aftab Nadeem, Fatemeh Askarian, Sun Nyunt Wai, Mona Johannessen, Kristin Hegstad

**Affiliations:** ^1^Department of Medical Biology, Research Group for Host-Microbe Interactions, UiT The Arctic University of Norway, Tromsø, Norway; ^2^Umeå Centre for Microbial Research (UCMR), Umeå University, Umeå, Sweden; ^3^Department of Molecular Biology, Umeå University, Umeå, Sweden; ^4^Faculty of Chemistry, Biotechnology and Food Science, The Norwegian University of Life Sciences (NMBU), Ås, Norway; ^5^Norwegian National Advisory Unit on Detection of Antimicrobial Resistance, Department of Microbiology and Infection Control, University Hospital of North-Norway, Tromsø, Norway

**Keywords:** *Staphylococcus aureus*, transcriptomic analysis, small RNAs, tRNA, extracellular vesicle

## Abstract

Bacterial extracellular vesicles (EVs) have a vital role in bacterial pathogenesis. However, to date, the small RNA-cargo of EVs released by the opportunistic pathogen *Staphylococcus aureus* has not been characterized. Here, we shed light on the association of small RNAs with EVs secreted by *S. aureus* MSSA476 cultured in iron-depleted bacteriologic media supplemented with a subinhibitory dosage of vancomycin to mimic infection condition. Confocal microscopy analysis on intact RNase-treated EVs indicated that RNA is associated with EV particles. Transcriptomic followed by bioinformatics analysis of EV-associated RNA revealed the presence of potential gene regulatory small RNAs and high levels of tRNAs. Among the EV-associated enriched small RNAs were SsrA, RsaC and RNAIII. Our finding invites new insights into the potential role of EV-associated RNA as a modulator of host-pathogen interaction.

## Introduction

*Staphylococcus aureus* (*S. aureus*), a Gram-positive bacterium, is a frequent colonizer of anterior nares of the healthy human population. This bacterium can cause various infections ranging from minor superficial skin infections to severe life-threatening infections such as osteomyelitis, pneumonia, endocarditis, bacteremia and sepsis (Wertheim et al., 2005; Mccaig et al., 2006; Foster et al., 2014). The adaptation of diverse lifestyles and the ability to cause diseases is due to the fact that *S. aureus* harbors arsenals of virulence factors involved in adhesion, invasion and dissemination (Novick, 2003).

Small RNA (sRNA) are heterogeneous small-sized transcripts (50–500 nucleotides) expressed under stressful environmental conditions which play important roles in growth processes, metabolism, stress adaptation and virulence (Tomasini et al., 2014; Westermann, 2018). Prokaryotic sRNAs are often non-coding and mainly originate from intergenic regions (Wagner and Vogel, 2003). They generally form secondary structures such as hairpins and stem-loops (Wagner and Romby, 2015). There are varieties of techniques to identify and characterize sRNAs (Lagos-Quintana et al., 2001; Wang et al., 2009; Li et al., 2012). Mizuno et al. first reported sRNAs with regulatory functions in *Escherichia coli* in 1980's, and a decade later Novick et al. reported regulatory sRNAs in *S. aureus* (Mizuno et al., 1984; Novick et al., 1989). Currently, there are about 250 sRNAs discovered in various strains of *S. aureus* grown under different experimental conditions, and the biological functions of most of them are yet to be determined (Guillet et al., 2013; Hermansen et al., 2018). Still, novel sRNA transcripts are reported from *S. aureus* strains, and the number is increasing with the advancement of high-throughput sequencing technology as well as robust computational methods (Liu W. et al., 2018; Westermann, 2018).

Extracellular Vesicles (EVs) are nanosized-proteolipids, with a spherical shape that are heterogeneous in size ranging from 50 to 500 nm (Askarian et al., 2018). Sometimes fusion of vesicles have resulted in formation of filamentous structures, also known as nanopods or nanotubes (Dongre et al., 2011; Dubey and Ben-Yehuda, 2011; Gill et al., 2018). EVs may contain virulence factors (Devos et al., 2015; Askarian et al., 2018; Wagner et al., 2018; Nadeem et al., 2020), such as toxins (Rivera et al., 2010; Coelho et al., 2019) as well as other enzymes (Smalley and Birss, 1987; Elhenawy et al., 2014), quorum sensing molecules (Mashburn and Whiteley, 2005; Brameyer et al., 2018; Morinaga et al., 2018) and nucleic acids such as DNA (Hagemann et al., 2014; Bitto et al., 2017; Langlete et al., 2019) and RNA (Sjöström et al., 2015; Koeppen et al., 2016; Choi J.-W. et al., 2017). Their content may vary depending on species and growth conditions (Bager et al., 2013; Ghosal et al., 2015; Koeppen et al., 2016). EVs might act as a decoy against antimicrobial peptides and phages (Manning and Kuehn, 2011), and are also involved in co-operation and/or competition with other pathogens (Lynch and Alegado, 2017; Choi et al., 2020). EVs can also influence biofilm formation and modulate host-immune responses (Manning and Kuehn, 2013; Schwechheimer and Kuehn, 2015; Liu Y. et al., 2018).

EVs from Gram-negative bacteria harbor sRNA involved in intra-species (microbe-microbe) (Whitworth, 2018) and inter-kingdom (microbe-host) interactions (Koeppen et al., 2016; Frantz et al., 2019) as well as pathogenicity (Song and Wai, 2009). However, scant functional and analytical data exist to support these claims in Gram-positive bacteria (Ghosal et al., 2015; Sjöström et al., 2015; Koeppen et al., 2016; Choi et al., 2018; Malabirade et al., 2018).

During infection, the availability of iron is strictly controlled by the host, and in order to survive pathogens must adapt their transcriptomic and metabolic pathways accordingly (Wilderman et al., 2004; Oglesby-Sherrouse and Murphy, 2013; Mäder et al., 2016). Nutrient limitation and antibiotics is furthermore known to increase vesiculation (Toyofuku et al., 2019). Subinhibitory concentrations of the last resort anti-staphylococcal antibiotic, vancomycin, has been shown to influence physiology, growth and toxin production by *S. aureus* (Hsu et al., 2011; Cafiso et al., 2012; He et al., 2017), and has been found to increase EV production in another Gram-positive species, *Enterococcus faecium* (Kim et al., 2019). Hence, in this study, we used iron-chelated media supplemented with vancomycin to evaluate whether the EVs produced by *S. aureus* MSSA476 are associated with sRNAs when grown in conditions that mimic an infection that is being treated with an antibiotic.

## Materials and Methods

### Strain and Growth Conditions

*S. aureus* subsp. *aureus* Rosenbach MSSA476 was purchased from LGC standard AB (ATCC- BAA-1721) (Sweden). The bacteria were grown at 37°C on BHI agar plate, BHI broth, or in trace metal-depleted BHI broth containing 0.5 μg/mL of vancomycin. The trace metals including divalent cations such as iron were lowered by treating the BHI broth with 2 g/L of chelex-100 resin (Bio-Rad, Californa, USA). The medium was subsequently filtered according to the manufacturer's instructions.

### Isolation of Bacterial Extracellular Vesicles

The bacterial EVs were isolated by a procedure described earlier (Askarian et al., 2018; Wagner et al., 2018), with slight modifications. A fresh overnight culture of the methicillin-susceptible *S. aureus* MSSA476 (1:100 dilution) was inoculated into 500 mL BHI (normal conditions) or Chelex-treated BHI broth containing 0.5 μg/mL of vancomycin (stressed conditions) at least two to five different days. The cultures were grown with shaking at 37°C for 16 h. The cultures were then centrifuged at 6,000 × g for 30 min. Bacterial pellets were discarded, and the supernatants was filtered through 0.2 μm filters (Millipore Express™ Plus, USA) and ultra-centrifuged at 100,000 × g for 3 h at 4°C in a 45 Ti rotor (Beckman, USA). EV pellets from each isolation were re-suspended in 500 μL RNAlater (Thermo Fisher Scientific, Massachusetts, USA) or in Phosphate-buffered saline (PBS) if EVs were to be used for microscopy or Nanoparticle Tracking Analysis, and kept at −80°C until further use. Prior to RNA isolation for RNA-seq, EVs from several isolations were thawed, pooled and concentrated using ultrafiltration (10 kDa Vivaspin 20, Sartorius, Germany). An overview of EV isolations from bacteria grown under stressed conditions and its downstream applications is provided in Supplementary Table 1.

### Transmission Electron Microscopy (TEM)

TEM was performed as described previously (Cavanagh et al., 2018; Wagner et al., 2018). Briefly, 5 μL of purified EVs were applied to Formvar coated 75 mesh hexagonal copper grids (Electron Microscopy Science, Pennsylvania, USA) and incubated for 5 min. Grids were washed with MQ water, and negatively stained with 2% methylcellulose and 3% uranyl acetate in a ratio of 9:1. The excess of stain was blotted away, and grids were then left to dry at room temperature. The samples were then visualized with a JEOL JEM 1010 transmission electron microscope (JEOL, Tokyo, Japan) operated at 80 kV.

### Atomic Force Microscopy (AFM)

The EVs were imaged by AFM, as described previously (Lindmark et al., 2009; Ahmad et al., 2019). Briefly, EVs were deposited onto a freshly cleaved mica surface (Goodfellow Cambridge Ltd., Cambridge, UK). Prior to imaging, EVs on mica were dried in a desiccator for about 2 h. Images were recorded on a Multimode 8 Nanoscope AFM equipment (Bruker AXS GmbH, Karlsruhe, Germany) using TappingMode. Images were gathered by NanoScope software using ScanAsyst in air with ScanAsyst cantilevers, at a scan rate of ~0.8–1.5 Hz. The final images were plane fitted in both axes and presented in a surface plot of the height mode.

### Nanoparticle Tracking Analysis (NTA)

The size distribution of EVs were determined using NanoSight NS300 (Malvern Instruments Ltd., Worcestershire, UK) equipped with CMOS camera and a blue laser module (488 nm, LM12 version C) (Jamaly et al., 2018). Briefly, EV samples were thawed and diluted (500×) in PBS to obtain a concentration within the recommended measurement range (1–10 × 10^8^ particles/mL). Using a 1 mL syringe, the sample was injected into the instrument and videos were captured in triplicate for 30 s. The mean values for size and concentration were analyzed using the NanoSight (NTA software, version 3.0).

### Labeling of Extracellular Vesicles

The EVs were stained using a previously described protocol with slight modifications (Nicola et al., 2009; Vdovikova et al., 2017). The vesicles were either untreated or treated with RNase (Roche diagnostics, Basel, Switzerland) to remove extracellular RNA then stained with lipid-specific dye, PKH2 or DiD (Sigma Aldrich) and subsequently with RNA specific dye SYTO RNASelect Green (Thermo Fisher Scientific, Massachusetts, USA). The stained vesicles were then ultra-centrifuged at a speed of 100,000 × g for 1 h at 4°C. The stained EVs were resuspended in PBS. Samples were mounted on a glass slide and examined by Leica SP8 inverted confocal system (Leica Microsystems) equipped with a HC PL APO 63 ×/1.40 oil immersion lens. Images were captured and processed using LasX (Leica Microsystems). Fluorescence intensity profiles were generated using the plot profile command in ImageJ-FIJI distribution (Schindelin et al., 2012) For quantification, EVs from 8 randomly selected fields (180 μm^2^) were counted. Results were pooled from two independent experiments and data are expressed as percentage.

### Bacterial Growth Curve and Viability Assay

A single colony of MSSA476 was inoculated into two 5 mL of BHI broth and grown overnight with shaking at 37°C. The 5 mL cultures were used to inoculate 500 mL BHI (normal) and 500 mL iron depleted BHI containing antibiotics (stressed). The cultures were incubated with shaking at 37°C, and optical density was measured every 30 min for 16 h. For the viability assay, 500 μL of each culture of bacteria grown under normal and stressed conditions for 16 h, were harvested. Viable plate count was carried out by plating 20 μL of 10-fold serial dilutions (from 10^−5^ to 10^−10^) on blood agar plates, which were incubated for 24 h at 37°C. Dilutions containing 10–100 colonies were counted, and the concentration was calculated as CFU/mL.

### Live and Dead Count

Bacterial cultures grown for 16 h in BHI (normal) and iron depleted BHI containing antibiotics (stressed) were analyzed for live and dead cells using LIVE/DEAD BacLight bacterial Viability and Counting Kit (Thermo Fisher Scientific, Waltham, USA) and BD LSRFortessa flow cytometer. Each bacterial culture was diluted 1,000-fold in 1 mL 0.85% filtered NaCl, which contain 0.5 μL SYTO 9, 2.5 μL Propidium iodide (PI), and 10 μL beads of size 6 μm. Beads had a concentration of 1 × 10^8^/mL and were diluted 100-fold. Cells were stained for 10–15 min at room temperature. Stained bacteria were analyzed using BD LSRFortessa flow cytometer using a voltage of 600, 250, 400, and 800 for forward scatter (FSC), side scatter (SCC), AF488 and PI, respectively. All scales were set to logarithmic amplification with gain voltages of 300, 250 and 200 for FSC, SSC, and AF488, respectively. Data were recorded for 1,000 bead events. Total events were recorded and density of bacterial culture in terms of bacteria/mL was calculated as (numbers of events in bacterial region) × (dilution factor)/(numbers of events in the bead region × 10^−6^).

### Extraction of RNAs

The crude collection EVs was stored in RNAlater; which is a preservative compatible with RNA isolation and downstream applications such as RNA sequencing and Reverse transcription. The EVs were centrifuged using Vivaspin® ultrafiltration spin columns (cutoff 10000MWCO) at 5,000 RPM for 15 min. The concentrated EVs were treated with RNaseA (50 μg/mL) for 30 min at room temperature to degrade all forms of extracellular RNA. Thereafter, to stop the RNase activity, EVs were treated with 5 μL of RNase inhibitor (Applied Biosystems, Massachusetts, USA) for 15 min at 37°C. Then the small RNA from *S. aureus* EVs were isolated by miRNeasy kit (Qiagen, Hilden, Germany), according to the manufacturer's instruction. Trizol and Chloroform used during RNA isolation are sufficient to remove traces of RNAlater. The concentration of RNA was measured by Qubit HS kit, which quantify sample concentration ranging from 250 pg/μL to 100 ng/μL (Thermo Fisher Scientific, Waltham, USA), and the quality of RNA was assessed by Nanodrop (Thermo Fisher Scientific, Waltham, USA, USA). A 260/280 ratio of 1.8 or higher was considered optimal. In our RNA prep it was found to be 1.85. In order to evaluate whether the isolated RNA was extravesicular or associated with EVs, RNA concentration was measured on crude EVs, RNase treated EVs and finally in RNA isolated from the RNase-treated EVs.

### rRNA Depletion, Library Preparation, and Sequencing

The isolated EV-associated RNA was treated with Ribo-zero rRNA removal kit (Illumina, Munich, Germany) according to the manufacturer's instructions to reduce ribosomal RNA (rRNA). Thereafter, the concentration of RNA was measured using Experion RNA HighSense Chips (Bio-Rad Laboratories, Inc, USA). The depleted RNA was cleaned and concentrated using RNeasy MinElute Cleanup kit (Qiagen, Hilden, Germany) and RNA clean & concentrator-5 kit (Zymo Research, California, USA). The rRNA-depleted RNA was fragmented, and reverse transcribed into cDNA using high capacity cDNA reverse transcription kit (Applied Biosystems, California, USA), and sequenced on an Illumina NextSeq550 platform.

### RNA Preparation, cDNA Synthesis, and qPCR

qPCR was used to confirm the presence of the three enriched sRNAs (SsrA, RSaC, and RNAIII). The EV-associated RNA was isolated as described above and treated with DNase (ArcticZymes, Tromsø, Norway) before RNA integrity and quantity were measured both by NanoDrop and Qubit. cDNA was prepared by reverse transcription kit (Applied Biosystems) using 100 ng RNA. qPCR reactions were performed in technical duplicates for pooled EV samples using SYBR Green master mix (Applied Biosystem) with the following primer pairs: SsrA-F/R: CACTCTGCATCGCCTAACAG/ GCGTCCAGAGGTCCTGATAC, RsaC-F/R: CAAAGGAAAGGGGCATACAA/ ACGCCATTCCCTACACACTC, RNAIII-F/R: AGTTTCCTTGGACTCAGTGCT/ GGGGCTCACGACCATACTTA. To perform qPCR, briefly, 2 μL of cDNA was used as a template for each 20 μL reaction, which was carried out with 100 nM of primers. Cycling conditions were as follows: initial denaturation 10 min at 95°C, 40 cycles of 15 s at 95°C and 60°C for 1 min as annealing and elongation temperature. The data were treated and analyzed with the Applied Biosystems (7300 Real-Time PCR System) to determine the Ct.

### PCR and Sanger Sequencing

RT-PCR was carried out in a Thermal Cycler (Applied Biosystems, Foster City, CA) in order to verify the three PCR amplicons (SsrA, RsaC and RNAIII) by agarose gel and DNA sequencing. The PCR was performed in a 20 μL reaction, containing gene-specific primers mentioned above and DreamTaq Green PCR Master Mix (Thermo Fischer Scientific, USA) according to the manufacturer's instruction. One μL of cDNA was used as the template. The cycling conditions were performed as follows: after an initial denaturation step of 2 min at 95 °C, 40 cycles were performed for 30 s at 95 °C, 60 s at 60 °C, and 1 min at 72 °C. A final extension step for 10 min at 72 °C was used. PCR products were further separated on a 1% agarose gel, stained with GelRed and visualized using Syngen Gel Imaging (Bio-Rad Laboratories Inc, USA). The PCR product was cleaned using PCR Clean-Up Kit (Promega, Norway). Sequencing reactions were performed in using a BigDye Terminator version 3.1 kit (Applied Biosystems) according to the manufacturer's instructions with the same primers as for the real-time PCR assay. Sequencing was performed on an Applied Biosystems 3,130 × l genetic analyzer.

### Bioinformatics Analysis

The fastQ files obtained after paired-end sequencing was checked for quality using the Galaxy webserver (https://galaxy-uit.bioinfo.no). Bcl2fastq program supplied by Illumina was used to convert bcl files to fastQ files, which automatically trims the adapters and generates clean reads. The clean reads were aligned with the reference genome (MSSA476; GenBank accession no. NC_002953.3) using Bowtie 2 (Langmead and Salzberg, 2012). Mapping of the EV reads to the reference genome resulted in a Sequence Alignment Map file that was converted to a Binary Alignment Map (BAM) file. The BAM and its associated annotation files of the reference genome were loaded into Artemis where manual searches for sRNAs were performed. Visualization and manual inspection of reading coverage were conducted using Artemis version 1.0 (Rutherford et al., 2000). All the sRNAs are listed based on genomic coordinates provided from the bacterial small regulatory RNA repository BSRD (http://kwanlab.bio.cuhk.edu.hk/BSRD). The sRNAs identified from Artemis were run separately for Rfam search in Artemis to gather information about RNA families and RNA elements, including accession numbers. In addition, the Rockhopper tool (Tjaden, 2019) was used to identify transcripts and operons and to elucidate bacterial transcriptomes. Transcripts from Rockhopper were visualized (.wig files) in the Integrative Genomics Viewer (IGV) (Robinson et al., 2011).

## Results

### RNA Is Associated With *S. aureus*-Derived EVs

The MSSA476 bacterial growth in BHI (normal condition) or trace metal-depleted BHI supplemented with a subinhibitory concentration of vancomycin mimicking infection (stress condition) were compared and found to be similar at 16 h (Supplementary Figure 1). The viability of bacteria was evaluated by flow cytometry and colony-forming units (CFU) enumeration and showed similar viability which was above 99.6% (Table 1, Supplementary Figure 2).

**Table 1:** Summary of bacterial counts using flow cytometry and total plate count method.

**Sample**	**Flow cytometry data**			**Plate count (CFU)**
	**Bacterial count (bacteria/mL)**	**Live bacteria (%)**	**Dead bacteria (%)**	
*S. aureus* grown under normal condition	1.8 × 10^10^	99.9	0.1	3.0 × 10^10^
*S. aureus* grown under stress condition	8.8 × 10^9^	99.6	0.4	2.3 × 10^10^

**Figure 1:**
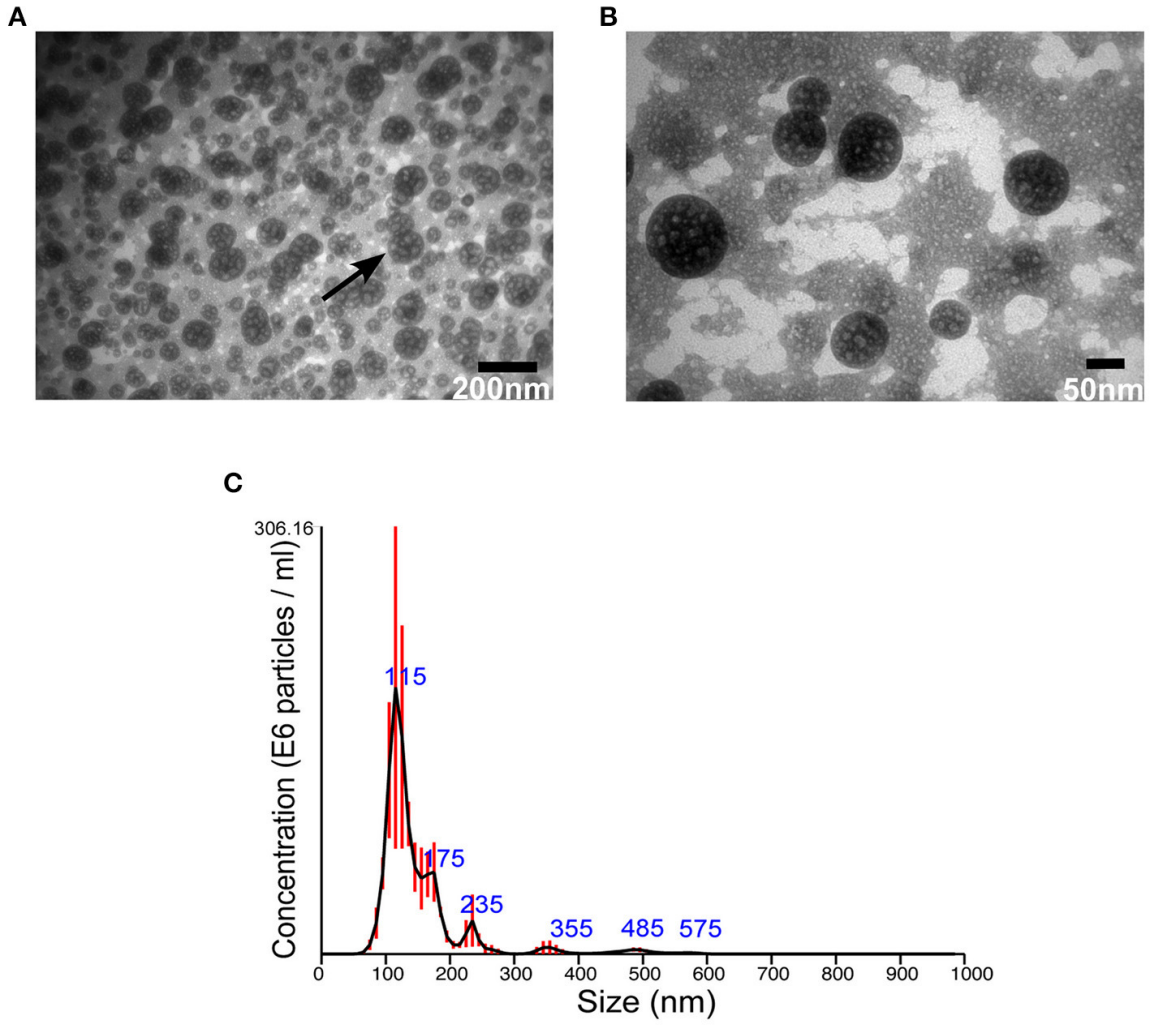
*S. aureus* MSSA 476 grown under infection mimicking condition produces spherical EVs of various sizes. **(A)** Low and **(B)** high magnification TEM images of crude EVs isolated from bacteria grown in iron-depleted BHI supplemented with subinhibitory concentration of vancomycin (black arrow indicates vesicles) **(C)** NTA showing total number and size distribution of EVs (mean in nm ± SD) isolated from *S. aureus* MSSA476. E6 particles/ml is the standard NTA output, and means 10^6^ particles/ml.

Then, EVs were isolated from *S. aureus* grown for 16 h under normal or stressed conditions. EVs were obtained from bacteria grown under both conditions. However, the number of particles, as well as protein concentration, was increased when bacteria were stressed (Supplementary Figure 3). Unfortunately, the yield of sRNA obtained from EVs isolated bacteria grown under normal condition was too low for RNA-seq. Therefore, we focused the study on EVs isolated from stressed bacteria. The morphology of EVs was evaluated by AFM and TEM. Aligned with other studies on MSSA476 (Gurung et al., 2011; Askarian et al., 2018), EVs were spherical in shape, though minor fusions were observed (Figures 1A,B, Supplementary Figure 4). In addition, the size distribution of vesicles was measured using NTA which revealed that the sizes ranged from 20 nm to 200 nm, although the majority of vesicles are between 100 and 150 nm. The analysis also showed some particles with sizes above 200 nm (Figure 1C), which might be due to aggregation or fusion of EVs.

**Figure 2:**
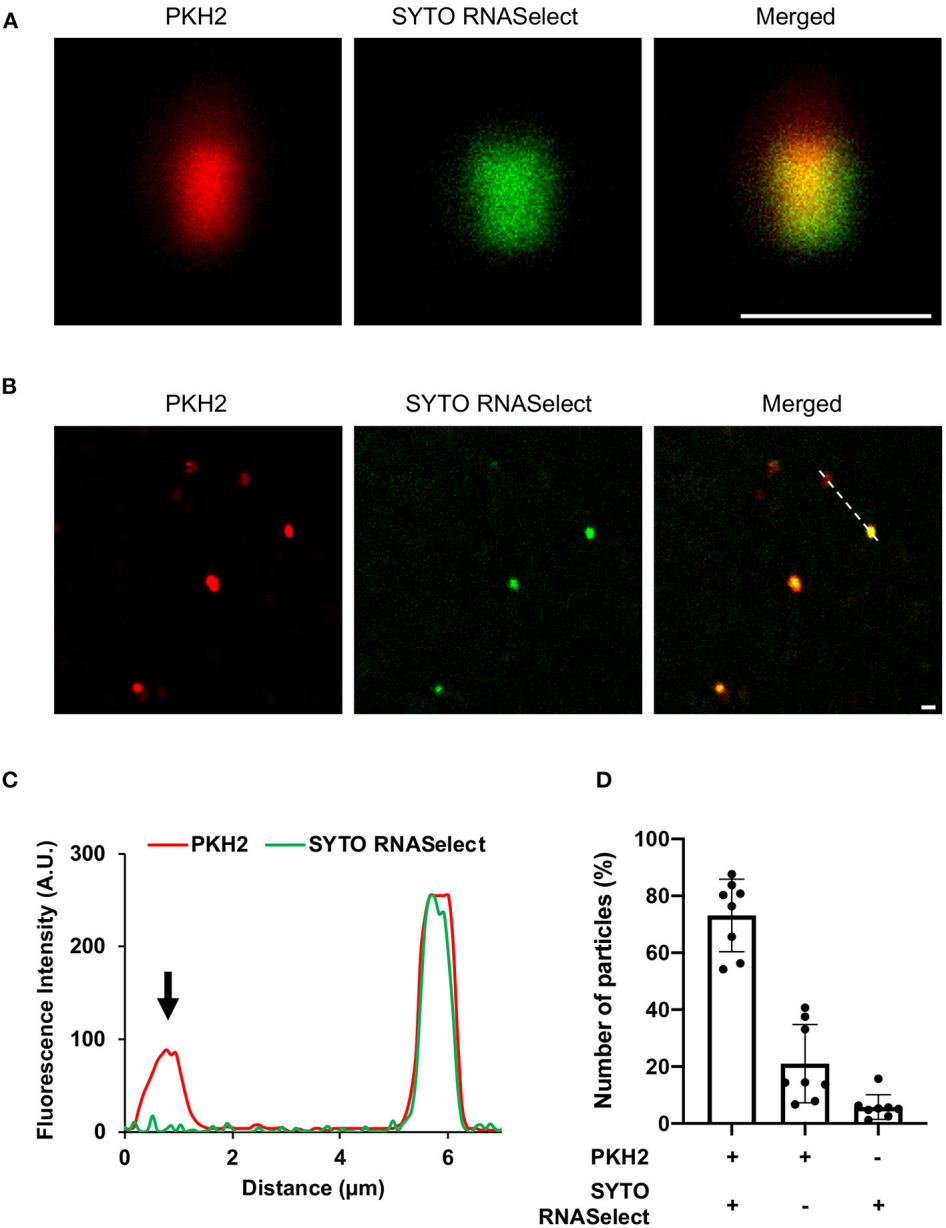
Confocal microscopy of **(A)** enlarged extracellular vesicle particles/aggregates and **(B)** multiple EV particles/aggregates from the field of view. The vesicles were stained with lipid specific dye, PKH2 (red) and RNA-specific dye, SYTO RNASelect (green). Scale bar, 1 μm [zoom 1 μm of **(A)**]. **(C)** Line graph showing fluorescence intensity profile of the dotted line across the extracellular vesicles in panel **(B)**. Arrowhead indicates absence of SYTO RNASelect fluorescence in the PKH2 stained extracellular vesicle. **(D)** Quantification of RNA and lipid positive particles. Data points from two different experiments and 8 fields of view.

Next, we wanted to evaluate whether RNA is associated with the EVs. The EVs were left untreated or treated with RNase to remove extravesicular RNA, and thereafter stained with RNA specific dye (green). EVs are known to contain lipids (Ghosal et al., 2015), and were therefore stained with a lipid-specific dye (red). The RNA and lipid-stained particles, which we assumed to be aggregated EVs (Ter-Ovanesyan et al., 2017), were then analyzed by confocal microscopy. As seen in Figures 2A,B and Supplementary Figures 5, 6, RNA and lipid stain co-localized in the majority of EVs. To confirm the sensitivity of the method, the fluorescence intensity (Figure 2C) was determined in co-stained and only lipid stained EVs indicated with dotted line (Figure 2B). Finally, we quantified co-localization of RNA and lipid particles by counting 8 random microscopic fields in both untreated and RNase-treated EVs. In RNase- treated EVs, we observed ~70% co-localization of RNA and lipid stained particles, while ~20% percent of the lipid stained particles were without any RNA stain (Figure 2D, Supplementary Figure 6B). Approximately 20% of the particles were only RNA-stained in untreated EVs, while only 6% of the RNase-treated EV particles were stained by RNA-specific stain only, supporting the efficacy of RNase treatment (Figure 2D, Supplementary Figure 6B). This low level of RNA stained particles in RNase-treated EVs might represent either non-specific aggregations of RNA dye, or a leakage of RNA from broken EVs during sample preparation, or the presence of small amounts of extra-vesicular RNA even after RNase treatment (Figure 2D).

**Figure 3:**
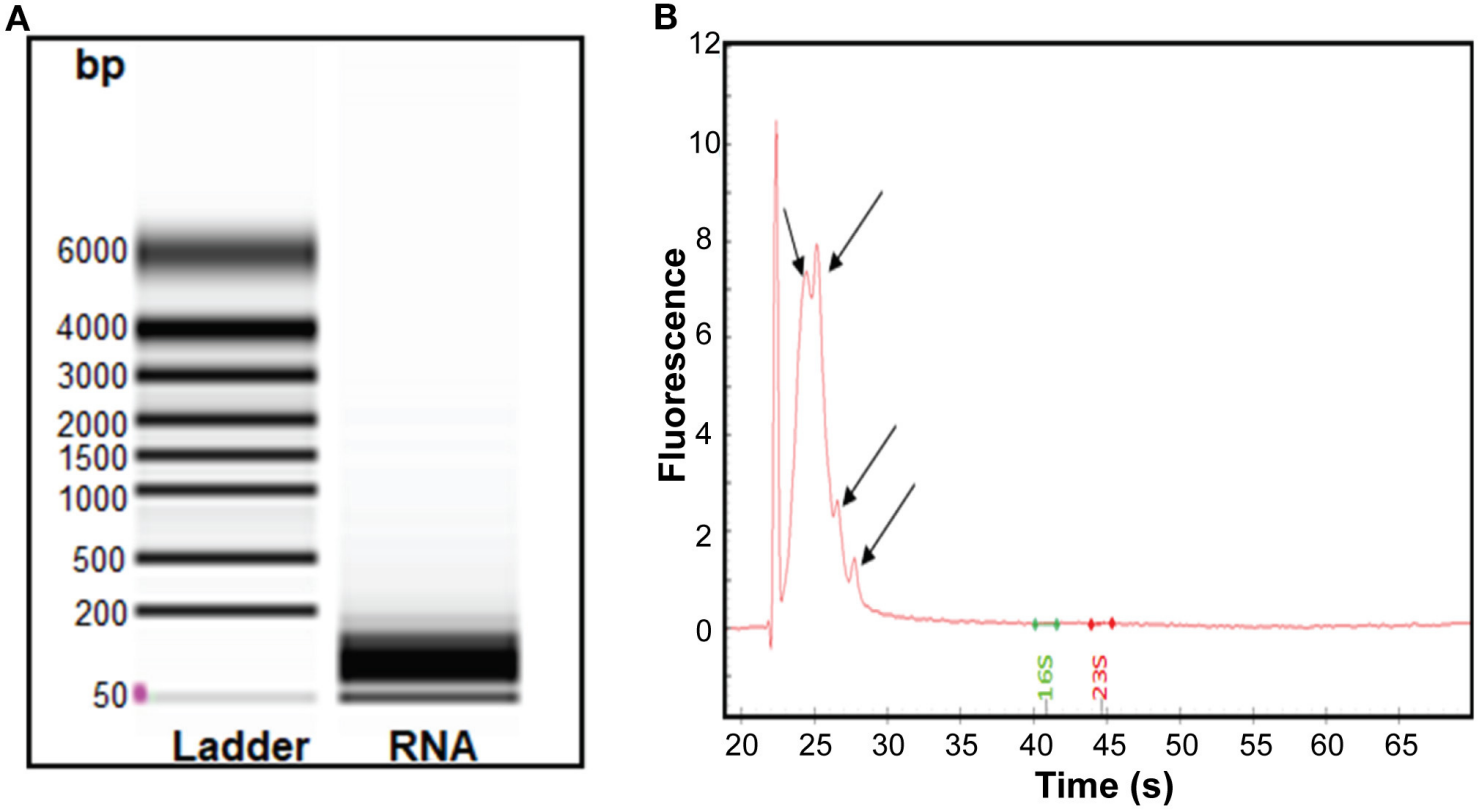
Analysis of the sRNA isolated from *S. aureus* EVs. **(A)** Virtual gel like image from bioanalyser. L, Ladder and lane 2 shows the RNA of the EVs. **(B)** Electropherogram displaying sRNA. The first peak in the electropherogram represent lower marker that is used as an alignment to RNA ladder. Other four small peaks appearing at the interval of migration time 24–28 s represent sRNA.

Having found that RNA was associated with RNase-treated EVs, we isolated sRNA which was analyzed further by bioanalyser. A smear of RNA in size range 50–200 bp was seen (Figure 3A). The obtained RNA was treated using the rRNA depletion kit, which reduced the average concentration of RNA from 77 to 40 ng/μL. Further analysis of the rRNA-depleted samples using bioanalyser revealed appearance of four peaks (Figure 3B, black arrows**)** at 24–28 s, known to be typical peak for <200 bp sRNA. Of note, no strong peaks appeared for ribosomal RNA (16S and 23S), indicating efficiency of sRNA enrichment by the miRNA kit and subsequent depletion of rRNAs. Hence, our data demonstrated that sRNA are associated with *S. aureus* EVs.

### tRNAs and sRNAs Were Enriched in *S. aureus* Derived EVs

The transcriptome profiling of the sRNA content in EVs was performed using RNA-seq analysis. Paired-end sequencing resulted in ~458,000 reads with lengths varied from 35 to 151 nucleotides. Eighty-seven percentage of the reads were aligned and found to be well-distributed over the reference genome of *S. aureus* strain MSSA476 (GenBank accession no. NC_002953.3) (Supplementary Table 2 - mapping statistics) (Supplementary Figure 7). The majority of reads corresponded to protein encoding RNAs, while 4 and 3.5% corresponded to rRNAs and tRNAs, respectively, 0.5% of the reads obtained by RNA-seq corresponded to sRNAs (Supplementary Figure 8). The sRNA reads corresponded to sRNAs with size distribution from 20 to 500 nt (Supplementary Figure 9).

A total of 62 RNAs, 276 5′ untranslated regions (5′ UTRs) and 276 3′ untranslated regions (3′ UTRs) were detected. Similarly, further aligning of the reads against *S. aureus* MSSA476 plasmid pSAS resulted in detection of five RNAs, seven 5′ UTRs, and 11 3′ UTRs (Supplementary Table 2 - Summary Rockhopper output). Next, operons in the *S. aureus* genome were defined as regions with continuous coverage of whole transcript reads by RNA-seq. This resulted in identification of 486 multi-gene operons consisting of 2–18 genes (for a total of 1,415 genes) (Supplementary Table 2 - operon Rockhopper output). Phage RNAs, e. g, transcripts encoding terminase subunits, tail proteins and portal protein, were detected among the protein encoding RNAs (Supplementary Table 2 - Rockhopper transcript output). Since our focus is on sRNAs, we chose to further describe only the tRNA and sRNA content.

Coverage of tRNA upstream of a regulatory region is shown in Supplementary Figure 10. The read counts of tRNAs in EVs varied from 4 to 984, with cove scores from 36.67 to 101.60 (Supplementary Table 2 - tRNAs in EVs). The most enriched tRNAs includes tRNA for Met, Asp, Leu, Tyr, Ser, Thr, Gly, and Phe (Table 2).

**Table 2:** The most enriched MSSA476 EV-associated tRNAs based on read counts.

**Element**	**Genomic coordinates**	**Full name (anticodon)**	**Cove score**	**Read count**	**GC content**	**Bases of selection**
tRNA	1937102.1937175	tRNA Met (CAT)	75.92	984	62.16	CGCGGGATGGAGCAGTTCGGTAGCTCGTCGGGCTCATAACCCGAAGGTCGGTGGTTCAAATCCGCCTCCCGCAA
tRNA	1937017.1937092	tRNA Asp (GTC)	83.32	859	61.84	GGTCTCGTAGTGTAGCGGTTAACACGCCTGCCTGTCACGCAGGAGATCGCGGGTTCGATTCCCGTCGAGACCGCCA
tRNA	533919.534007	tRNA Leu (TAA)	75.33	607	61.80	GCCGGGGTGGCGGAACTGGCAGACGCACAGGACTTAAAATCCTGCGGTGAGAGATCACCGTACCGGTTCGATTCCGGTCCTCGGCACCA
tRNA	1937971.1938059	tRNA Leu (TAA)	76.44	590	61.80	GCCGGGGTGGCGGAACTGGCAGACGCACAGGACTTAAAATCCTGCGGTGAGTGATCACCGTACCGGTTCGATTCCGGTCCTCGGCACCA
tRNA	1936757.1936837	tRNA Tyr (GTA)	69.29	525	61.73	GGAGGGGTAGCGAAGTGGCTAAACGCGGCGGACTGTAAATCCGCTCCTTCGGGTTCGGCAGTTCGAATCTGCCCCCCTCCA
tRNA	1937190.1937282	tRNA Ser (TGA)	74.09	829	61.29	GGAGGAATACCCAAGTCCGGCTGAAGGGATCGGTCTTGAAAACCGACAGGGCCTTAACGGGCCGCGGGGGTTCGAATCCCTCTTCCTCCGCCA
tRNA	1937407.1937496	tRNA Ser (TGA)	61.04	657	60.00	GGAGGAATACCCAAGTCCGGCTGAAGGGATCGGTCTTGAAAACCGACAGGGGCTTAACGGCTCGCGGGGGTTCGAATCCCTCTTCCTCCG
tRNA	1936843.1936918	tRNA Thr (TGT)	92.93	560	55.26	GCCGGCCTAGCTCAATTGGTAGAGCAACTGACTTGTAATCAGTAGGTTGGGGGTTCAAGTCCTCTGGCCGGCACCA
tRNA	533837.533911	tRNA Gly (GCC)	86.82	518	54.67	GCAGAAGTAGTTCAGCGGTAGAATACAACCTTGCCAAGGTTGGGGTCGCGGGTTCGAATCCCGTCTTCTGCTCCA
tRNA	1936926.1936998	tRNA Phe (GAA)	76.42	635	50.68	GGTTCAGTAGCTCAGTTGGTAGAGCAATGGATTGAAGCTCCATGTGTCGGCAGTTCGACTCTGTCCTGAACCA

*See Supplementary Table 2 for the full list of tRNAs*.

The 67 sRNAs identified by Rockhopper software were manually checked with Artemis and 49 sRNAs were validated using the Rfam database. The MSSA476-derived EVs carried several sRNAs with read counts varying from 1 to 80 (Supplementary Table 2 - small RNA in EVs). 6S RNA and SsrA showed the highest read counts of 80 and 65, respectively (Table 3, read density of SsrA, 6S RNA, RNAIII, and RsaC are shown in Supplementary Figure 11).

**Table 3:** The most enriched MSSA476 EVs- associated small RNAs ranked based on read counts^a^.

**Element**	**RFAM accession**	**Description**	**Functions**	**Genomic coordinates**	**Gene length (nt)**	**Strand^b^ (F/R)**	**Read count^c^**	**Bit score**	**GC content (%)**
6S RNA	RF00013	Protein-binding small RNA	Involved in antibiotic resistance (Lalaouna et al., 2014)	1685656..1685846	197	R	80	97.5	42.41
SsrA RNA	RF00023	Protein-binding small RNA	Rescues stalled ribosomes during translation of defective mRNAs and biosynthesis of pigment (Liu et al., 2010; Guillet et al., 2013)	837496..837857	362	F	65	162.6	43.92
RsaC	RF0188	Trans-encoded antisense RNA	Oxidative stress and metal-dependent nutritional immunity (Lalaouna et al., 2019)	673626..674066	441	R	33	555.6	35.37
T-box	RF00230	Regulatory elements	Involved in amino acid metabolism (Schoenfelder et al., 2013)	1674489..1674687 385924..386088 386093..386293 1199542..1199714 12486..12696	178 165 201 173 211	R F R F F	32 24 22 16 13	90.55 96.6 114.2 80.94 93.3	34.27 31.52 39.8 34.10 33.65
4.5S RNA	RF00169	Trans-encoded antisense RNA	Processing of tRNAs (Szafranska et al., 2014)	485461..485730	270	F	24	69.1	48.52
FMN riboswitch	RF00050	Regulatory element	Controls expression of de novo riboflavin	1551305..1551439	135	R	23	121.7	46.67
SAM riboswitch	RF00162	Regulatory element	Involved in amino acid (methionine) metabolism	2372869..2372964	96	R	21	78.2	47.92
fstAT	RF01797	Trans-encoded antisense RNA	Type I toxin-antitoxin system that interfere with bacterial membrane (Schuster and Bertram, 2016)	1873399..1873493	95	R	19	90.2	42.11
rli28	RF01492	Trans-encoded antisense RNA	Role in virulence (Romby and Charpentier, 2010)	2205592..2205772	181	R	19	123.8	34.25
L19_leader	RF00556	Regulatory element	NA	1254259..1254301	43	F	18	50.0	41.86
yjdF	RF01764	Regulatory element	Regulate gene expression upon binding with heterocyclic aromatic compounds (Li et al., 2016).	423980..424080	101	F	17	92.9	38.61
rli28	RF01492	Trans-encoded antisense RNA	Role in virulence (Romby and Charpentier, 2010)	2030445..2030623	179	R	17	85.6	37.43
TPP riboswitch	RF00059	Regulatory element	Involved in biosynthesis and transport of thiamine (Sudarsan et al., 2005)	2155664..2155766	103	F	16	70.9	40.78
T-box leader	RF00230	Regulatory element	Involved in amino acid metabolism	1791148..1791366	203	R	15	80.52	31.53
RNAIII	RF00503	Trans-encoded antisense RNA	Involved in virulence (hemolysins) (Boisset et al., 2007)	2086458..2086973	516	R	14	472.2	28.68
RsaJ	RF01822	Trans-encoded antisense RNA	NA	2479454..2479740	287	F	13	332.1	30.66
Lysine riboswitch	RF00168	Regulatory element	Regulate expression of lysine biosynthesis and transport genes (Blount et al., 2007)	1732415..1732590	176	F	12	104.6	31.82
fstAT	RF01797	Trans-encoded antisense RNA	Type-I toxin-antitoxin systems (Blenkiron et al., 2016)	2483979..2484075	97	F	12	78.12	40.21
L10_Leader	RF00557	Regulatory element	NA	566720..566866	127	F	10	86.8	31.21

*See Supplementary Table 2 for the full list of sRNAs*.

a *The cutoff value was assigned as >10*,

b Direction of strand alignment, F, Forward; R, Reverse;

c *The total number of sRNA sequence reads. NA, Not available*.

### Validation of *S. aureus* SrrA, RsaC, and RNAIII RNA Associated With EVs

**Figure 4:**
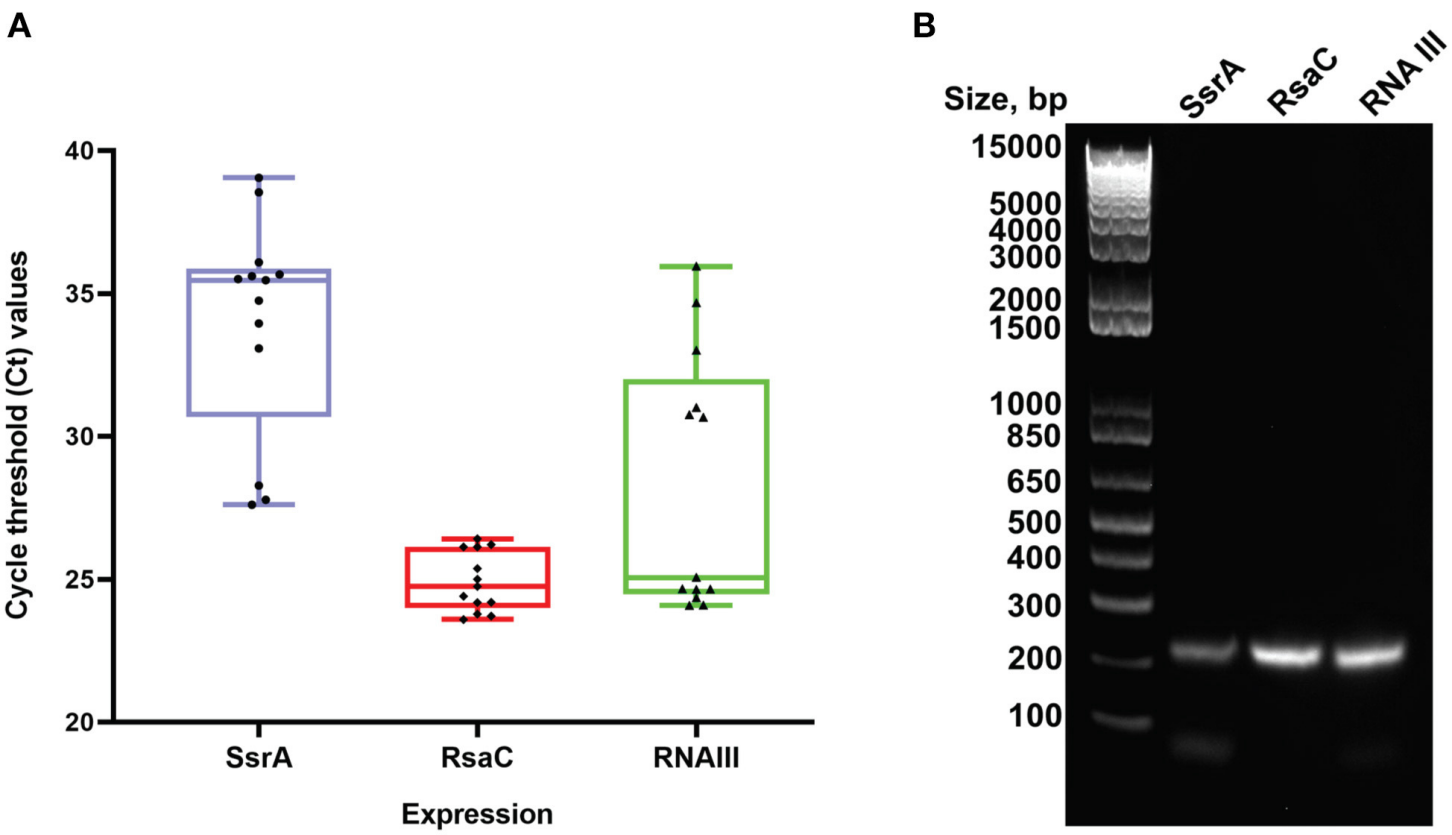
Real Time PCR validation analysis. **(A)** Boxplot representation of Ct values for SsrA, RsaC and RNAIII isolated from *S. aureus* EVs. Boxes represent interquartile range, central line is median, Whiskers are upper and lower adjacent values and Dots represent number of data points. **(B)** Agarose gel electrophoresis of the RT-PCR products obtained from RNA isolated from RNase-treated EVs. The expected amplicon sizes for SsrA, RsaC, and RNAIII are 204, 204, and 202 bp, respectively. The left lane shows molecular weight markers.

Among the enriched sRNA were SsrA, RsaC, and RNAIII (Table 3). SsrA and RsaC RNAs are involved in antibiotic resistance through their modulation of RNA fate and protein activity (Lalaouna et al., 2014), while RNAIII, not only have regulatory function but also encode 26 amino acid long δ-toxin (Novick et al., 1993; Caldelari et al., 2013). To validate our results obtained from transcriptomic analyses, we performed qPCR on RNA obtained from EVs using primers targeting SsrA, RsaC and RNAIII. The results presented in boxplot (Figure 4A) are based on three biological repeats (Supplementary Table 3) which confirmed presence of these three transcripts associated with EVs. The presence was finally confirmed by PCR of cDNA yielding DNA fragments of expected sizes (Figure 4B), and by Sanger sequencing which confirmed the identity of *ssrA, RsaC*, and *RNAIII* (Supplementary Figure 12).

## Discussion

*S. aureus* harbors a multitude of virulence factors that are tightly regulated during infection. Several studies have shown that exposure to sub-MIC antibiotic concentrations enhances *S. aureus* ability to adapt to physiological changes, survive and persist in human hosts (Kaplan et al., 2012; Howden et al., 2013). One of the mechanisms modulating virulence and pathogenicity of *S. aureus* is via the release of EVs (Gurung et al., 2011; Thay et al., 2013; Askarian et al., 2018; Schlatterer et al., 2018; Andreoni et al., 2019). Virulence factors such as hemolysin, loaded as vesicular cargo, are delivered to host cells via fusion of vesicles with the host cholesterol-rich membrane. In addition, *S. aureus*-derived EVs also release lipoproteins, which play a significant role in modulating TLR2 activation and are involved in pathogenesis. In general, various pathophysiological functions ranging from cellular inflammation to host cell death could be mediated by *S. aureus* EVs (Hong et al., 2011; Kim et al., 2012).

The first report of RNA associated with EVs was published in 1989 in Neisseria gonorrhoeae (Dorward and Garon, 1989). Henceforth, many intensive studies have been conducted to characterize RNAs and their functions (Scanlan, 2014; Sjöström et al., 2015; Blenkiron et al., 2016; Koeppen et al., 2016; Choi et al., 2018; Malabirade et al., 2018).

A crude EV pellet was used as the source material to enable isolation of sufficient sRNA for sequencing. The crude EV pellet was further concentrated using ultrafiltration columns with cutoff of 10 kDa, which remove lipoprotein aggregates (Ramirez et al., 2018). The crude EVs were RNase treated to remove any RNA that is not associated with EVs, before sRNA was isolated for high-throughput RNA sequencing. Adequately replicated RNA quantification isolated from EVs and RNase-treated EVs confirmed a reduction in RNA from RNase treatment (Supplementary Table 1). Since staining of RNase-treated EVs with RNA and lipid dyes showed RNase-mediated reduction in particles that were only RNA stained, the ~70% co-localization of RNA and lipid particles were assumed to be EVs (Figure 2, Supplementary Figures 5, 6). We could isolate sRNA from RNase-treated EVs, and thus we concluded that sRNA is associated with EVs. RNA-seq data revealed that SsrA, RsaC, and RNAIII were among the most enriched sRNAs associated with the EVs. To validate our findings, we repeated isolation of sRNA in triplicates from RNase treated EVs and confirmed presence SsrA, RsaC, and RNAIII by qPCR, conventional PCR and Sanger sequencing of the PCR products (Figure 4, Supplementary Table 1).

We also identified phage-like sequences in EVs by RNA-seq (Supplementary Table 2). The presence of these might be due to vancomycin-induced activation of one or two of the prophages harbored by *S. aureus* MSSA476 (Holden et al., 2004), which then pelleted with the vesicles. This agrees with others, who also obtained phage or phage tail particles in EVs when bacteria were exposed to antibiotics (Kharina et al., 2015; Devos et al., 2017; Andreoni et al., 2019). One of the limitations of using the crude pellet as the source of EVs for isolation of RNA is that it not only contains EVs, but also other nanoscale contaminants (e.g., the filaments and bacteriophages). We assumed that the source of RNA, in our study, is predominantly from RNase-treated EVs, but it is conceivable that some sequences are from other nanoscale contaminants pelleted along with the EVs, in a form that is protected from RNase, and at a concentration or of a size that is not visible by the fluorescence microscopy.

In bacteria, 16S and 23S rRNA are the most abundant RNAs that accounts for more than 90% of the total RNA biotype (Petrova et al., 2017). The abundance of rRNA reduces the sequencing depth for other RNA classes, thus an rRNA depletion strategy was implemented to ensure sufficient coverage of the transcriptome from bacterial RNA-seq data. Although in our study, fragments of mRNA were most abundant, we focused on characterizing sRNA, given they play important roles in EV biogenesis and virulence (Diallo and Provost, 2020; Lécrivain and Beckmann, 2020).

The observed EV sizes agreed with other studies from *S. aureus* (Gurung et al., 2011; Askarian et al., 2018; Wang et al., 2018; He et al., 2019). Antibiotics and other stressful conditions are considered as trigger factors for EV formation (Maredia et al., 2012; Prados-Rosales et al., 2014; Andreoni et al., 2019). In agreement with this, we observed a higher yield of EVs when the bacteria were grown in iron-limited media supplemented with vancomycin compared to typical bacteriologic media (Supplementary Figure 3).

Since we inoculated iron-chelated BHI media with an overnight grown inoculum in 1:100 dilutions, there is the possibility of transfer of trace amounts of iron. However, it has been shown that *S. aureus* utilizes a large proportion of iron within 6 h of aerobic growth in tryptic soy broth media (Ledala et al., 2014). Iron utilization by *S. aureus* in BHI might be similar. In addition, the traces of iron transferred through the inoculum would have been utilized by growing *S. aureus*, leaving media chelated for iron after a 16 h incubation (Ledala et al., 2010 and Ledala et al., 2014). It will, however, be interesting in the future to explore other culture media, such as RPMI 1640, which may better reflect infection conditions (Dauros-Singorenko et al., 2017).

Although there are multiple studies in Gram-positive bacteria showing altered sRNA expression due to antibiotic treatment (Felden and Cattoir, 2018; Gao et al., 2020), few studies have evaluated RNA content associated with EVs upon antibiotics exposure. Exposure to antimicrobials such as ciprofloxacin, tetracycline and melittin treatment of *Acholeplasma laidlawii* resulted in very variable numbers of sequence reads of predominantly 14–60 nt RNAs associated with EVs. In addition, tRNA fragments (mainly tRNA-Leu, tRNA-Arg, tRNA-Asn, and tRNA-Met) were predominant (Chernov et al., 2018) which is in line with our study. There are also few reports in eukaryotic exosomes and protozoal EVs confirming that exosome RNA levels altered by cellular stress (Bayer-Santos et al., 2014).

In general, RNAs are unstable and prone to degradation by RNase present in the extracellular milieu. However, recent reports indicate that RNAs encapsulated in EVs are protected from degradation by the exogenous RNase (Weber et al., 2010; Dauros-Singorenko et al., 2018), which support our results where we could isolate RNA from the RNase-treated EVs. Our RNase treatment of EVs would also facilitate degradation of eventual contaminating RNA that might passively have been released from the 0.4% dead cells in the culture used for vesicle isolation. Nevertheless, some of the RNA-protein complex sticking to the EVs might still have been protected from RNase treatment (Ramirez et al., 2018).

Transcriptome analysis of the EVs revealed the presence of tRNAs and sRNAs (Tables 2, 3 and Supplementary Table 2). Based on the read counts, the tRNA fragments were found to be most abundant. Abundant reads of tRNA fragments have previously been found in EVs released by bacteria (Ghosal et al., 2015; Koeppen et al., 2016), fungi (*Paracoccidiodes brasiliensis, Histoplasma capsulatum*) (Da Silva et al., 2015; Alves et al., 2019) and protists (*Trypanosoma cruzi, Leishmania* species) (Garcia-Silva et al., 2014; Lambertz et al., 2015). Interestingly, EVs of *Pseudomonas aeruginosa* also contained tRNA-Met fragments which entered the host cell and inhibited IL-8 secretion, which are considered as a chemoattractant of neutrophils (Koeppen et al., 2016).

There has been discussed whether RNAs associated with EVs are “intact,” fragmented or specifically processed products. EVs have been found to be associated with various fragments derived from mRNAs, rRNA and tRNA (Mateescu et al., 2017), which is in agreement with our study. Due to the small size of vesicles (20–200 nm), we might speculate that inside EVs, mostly smaller fragmented RNAs should be enriched. However, we cannot exclude the possibility of having full length RNA, given Buck and colleagues reported full length YRNAs exclusively inside Nematode-derived EVs (Buck et al., 2014). In our case, we see fragment lengths of 35–150 nt.

It has been reported that sRNA present in Vibrio and other Gram-negative bacteria play a role in vesicle biogenesis (Song et al., 2008; Choi H.-I. et al., 2017). MicA from *E. coli* induce EV biogenesis. Likewise, Song and collaborators also identified VrrA, a homolog of *E. coli* MicA in *Vibrio cholera* that controls EV formation and contributes to bacterial fitness in certain stressful environments (Song et al., 2008). Besides sRNAs, Sle1, an autolysin, has been shown to facilitate vesicle biogenesis (Wang et al., 2018).

The presence of EV-associated sRNA (SsrA, RsaC, and RNAIII) was confirmed by RNA-seq, qPCR, RT-PCR, and sequencing of obtained replicons. Among these, SsrA is involved in defective mRNAs decay, rescue of stalled ribosomes, support of phage growth, and modulation of the activity of DNA binding proteins (Karzai et al., 2000; Janssen and Hayes, 2012). Earlier reports have shown an increase of SsrA RNA in *Streptococcus pyogenes* and *Helicobacter pylori* in the presence of antibiotics (Steiner and Malke, 2001; Thibonnier et al., 2008). In our study, the high coverage of SsrA RNA associated with *S. aureus* EVs might be due to the use of vancomycin stress prior to vesicle isolation. 6S RNA plays an important role in cell survival and persistence during the stationary phase (Wassarman and Storz, 2000; Trotochaud and Wassarman, 2004) and was also found associated with the EVs. Interestingly, RNAIII (Table 3), which has major roles in virulence and pathogenicity (Boisset et al., 2007; Toledo-Arana et al., 2007), was also associated with EVs of *S. aureus*. The validation of RNAIII associated with EVs of *S. aureus* opens further study on the possibility of sRNA-mediated interspecies communication, as RNAIII has already been proved to be involved in the regulation of quorum sensing communication systems to coordinate the expression of virulence factors (Diallo and Provost, 2020; Lécrivain and Beckmann, 2020). Indeed, EVs could be used as communication vehicles only if they could transfer associated RNA into host cells and have a functional effect. Another possibility is that bacteria utilize EVs to eliminate unwanted RNAs, including sRNA and tRNA fragments (Groot and Lee, 2020).

Importantly, EVs influence *S. aureus* virulence over the course of systemic infection (Askarian et al., 2018). Recently, RNAs (circulatory/and or EV-associated) were considered as virulence factors due to their role in the infection process via multifaceted signaling pathways. The signaling pathways involved depend upon the delivery of bacterial RNA into the host cells. It has been shown that bacterial RNA can be delivered to human cytosol (Vanaja et al., 2014) and the phagosomal compartment (Cervantes et al., 2013) and EV-associated RNA has been localized in the human cell nucleus of human bladder carcinoma cells (Blenkiron et al., 2016). The sRNAs associated with EVs found in this study has been shown to be involved in quorum sensing (Novick and Geisinger, 2008), oxidative stress (Lalaouna et al., 2019), antibiotic resistance and metabolism (Lalaouna et al., 2014). All these processes are important for virulence and modulation of bacterial pathogenicity. EVs have some striking similarities with exosomes that are secreted from most mammalian cell types. Exosomes are involved in transport of mRNAs and miRNAs from donor to recipient cells to modulate gene expression (Zhang et al., 2015; Lu et al., 2019). They have similar size (around 50–200 nm in diameter) and carry payloads of proteins, lipids, and genetic materials such as the bacterial membrane vesicles. Both types can deliver functional molecules to distant extracellular compartments and tissues.

Recently, it was described that eukaryotic sRNA profiles of serum exosomes derived from individuals with tuberculosis can facilitate the development of potential molecular targets for detection/diagnosis of latent and active tuberculosis (Lvu et al., 2019). In addition to eukaryotic sRNA in exosomes, circulating sRNA (ASdes) from *Mycobacterium tuberculosis* was found in patients suffering from active tuberculosis, implicating their role as diagnostic biomarkers (Fu et al., 2018). This makes us hypothesize that some of the sRNAs we have validated in EVs (RNAIII and SsrA) have a potential to be used as biomarkers for bloodstream infections (Bordeau et al., 2016), joint infections (osteomyelitis) (Deng et al., 2020), tissues infections (e.g., chronic biofilm infections) and/or bacterial persistence (Romilly et al., 2014; Schoenfelder et al., 2019). Identifying sRNAs as biomarkers should not be limited to pathogenic strains but also to nasal and other commensal strains.

A previous study compared RNA contents of group A streptococcal cells vs. their EVs, and found that some RNA species were differentially abundant (Resch et al., 2016). For future studies, it would be interesting to do similar studies in *S. aureus*, and also to compare whether media or antibiotics influence the EV cargo. Further investigation is also needed to address whether RNAs found inside the vesicles are entrapped during vesicle biogenesis or if there are some sorting of RNA into the vesicles.

In conclusion, to our knowledge, this is the first study describing sRNAs associated with *S. aureus* extracellular vesicles. Various tRNA and sRNA associated fragments with several biological or regulatory functions have been identified associated with the EVs. This study opens further questions concerning sorting mechanisms by which RNA can be packed inside EVs and their roles in host-microbe as well as microbe-microbe interactions. Targeting those sRNA may open avenues toward a novel anti-virulence strategy to treat intractable bacterial infections.

## Data Availability Statement

The datasets presented in this study can be found in online repositories. The names of the repository/repositories and accession number(s) can be found in the article/Supplementary Material.

## Author Contributions

BJ, MJ, and KH designed the experiments and prepared the manuscript. BJ performed the majority of the lab experiment. AN assisted in confocal microscopy and image analysis. KH assisted in bioinformatics analysis, while BS did flow cytometry, and assisted in qPCR experiments. FA and BS contributed to writing of the results-section. BJ, BS, FA, AN, SW, MJ, and KH gave intellectual input. All authors read and approved the final manuscript.

## Conflict of Interest

The authors declare that the research was conducted in the absence of any commercial or financial relationships that could be construed as a potential conflict of interest.

## Supplementary Material

Supplementary Figure 1 Growth curves of *S. aureus* grown under BHI (normal condition) and iron-depleted BHI media with subinhibitory concentration of vancomycin (stressed condition).

Supplementary Figure 2 Flow cytometry analysis of S. *aureus* grown in **(A)** BHI (normal condition) **(B)** iron-depleted BHI media with subinhibitory concentration of vancomycin (stressed condition) for 16 h. Side scatter is represented on X-axis and AF488 on Y-axis. Gates P1, P3, and P6 were set for beads, live cells and dead cells, respectively.

Supplementary Figure 3 Measurement of EV yield obtained from bacteria grown in BHI (normal condition) and iron-depleted BHI with subinhibitory concentration of vancomycin (stressed condition). **(A)** EV number quantified by NTA. **(B)** Protein concentration in EVs measured by Qubit (protein assay kit). The mean ± SD is shown. The results represent three biological repeats (individual EV isolations) (NTA and protein concentration measurements). ^*^*P* < 0.05, unpaired *t*-test.

Supplementary Figure 4 AFM image of EVs isolated from bacteria grown in iron-depleted BHI supplemented with subinhibitory concentration of vancomycin (White arrow indicates vesicles).

Supplementary Figure 5 Confocal microscopy on intact RNase-treated EV particles/aggregates stained with **(A)** lipid specific dye, DiD (red), and **(B)** RNA-specific dye, SYTO RNAselect (green). **(C)** The image shows the overlay observed under the microscope. The overall percentage of co-localization in one microscopic field was found to be 78.3% which is in line with the results using another lipid specific dye in Figure 2. The white rectangular box and lower panel highlights the magnified region in the inset. The scale bar is drawn to 7.5 μm.

Supplementary Figure 6 **(A)** Confocal microscopy of extracellular vesicles without RNase treatment stained with lipid specific dye, PKH2 (red) and RNA-specific dye, SYTO RNASelect (green). Arrowhead (white) indicates absence of PKH2 fluorescence for the particle, while arrowhead (blue) shows co-localization of PKH2 stained extracellular vesicle with SYTO RNASelect dye. Scale bar, 1 μm. **(B)** Arrowhead indicates absence of PKH2 fluorescence in the SYTO RNASelect stained particle. **(C)** Quantification of RNA and lipid positive particles. Data points from two different experiments and 8 fields of view.

Supplementary Figure 7 Transcriptome landscape of *S. aureus* EVs. RNA sequencing reads were mapped to reference genome (NC_002953) using Rockhopper v 2.0.3. The mapped RNAs, UTRs and, multi-gene operons were visualized in the IGV genome browser. The first red track corresponds to RNA transcripts. The blue track corresponds to UTRs of protein coding genes. The pink track corresponds to multi-gene operons. The final purple track at the bottom of the image corresponds to protein coding genes and RNA genes annotated in RefSeq.

Supplementary Figure 8 Distribution of EV RNAs showing percent of reads mapped to each RNA biotype in *S. aureus* chromosomes **(A)** and plasmid **(B)**, respectively.

Supplementary Figure 9 **(A)** Schematic representation of sRNA identification. **(B)** Size length distribution of sRNAs associated with EVs of *S. aureus*.

Supplementary Figure 10 Read density profiles of tRNAs (pink highlighted region) downstream of the PerR regulatory region visualized by Artemis genome browser. The abundant loci close to tRNAs are ribosomal RNA. The X-axis represent the position in the genome and the Y-axis represent the numbers of reads mapped (coverage) at that location.

Supplementary Figure 11 Artemis genome viewer windows showing read density profiles of **(A)** SsrA, **(B)** 6S RNA, **(C)** RNAIII, and **(D)** RsaC RNA (pink highlighted regions). X-axis represent the position in the genome and the Y-axis represent the numbers of reads mapped (coverage) at the specific location.

Supplementary Figure 12 Sanger sequencing alignment of SsrA, RsaC and RNAIII RNAs. Sequences were aligned using BioEdit alignment tool.

## Abbreviations

ATCC: American type culture collection
AFM: Atomic Force Microscopy
KEGG: Kyoto Encyclopedia of Genes and Genomes
IGV: Integrative Genomics Viewer
EVs: Extracellular vesicles
NTA: Nanoparticle Tracking Analysis
EVs: Extracellular Vesicles
rRNA: Ribosomal RNA
RNA-Seq: RNA Sequencing
sRNA: Small RNA
TEM: Transmission Electron Microscopy
tRNA: transfer RNA
UTRs: Untranslated regions.
