# Supplementary material for: Transcriptome Profiling of Staphylococcus aureus Associated Extracellular Vesicles Reveals Presence of Small RNA-Cargo
Source: Front Mol Biosci. 2021 Jan 13;7:566207. doi: 10.3389/fmolb.2020.566207 (PMC7838569; doi:10.3389/fmolb.2020.566207)

Figure S1

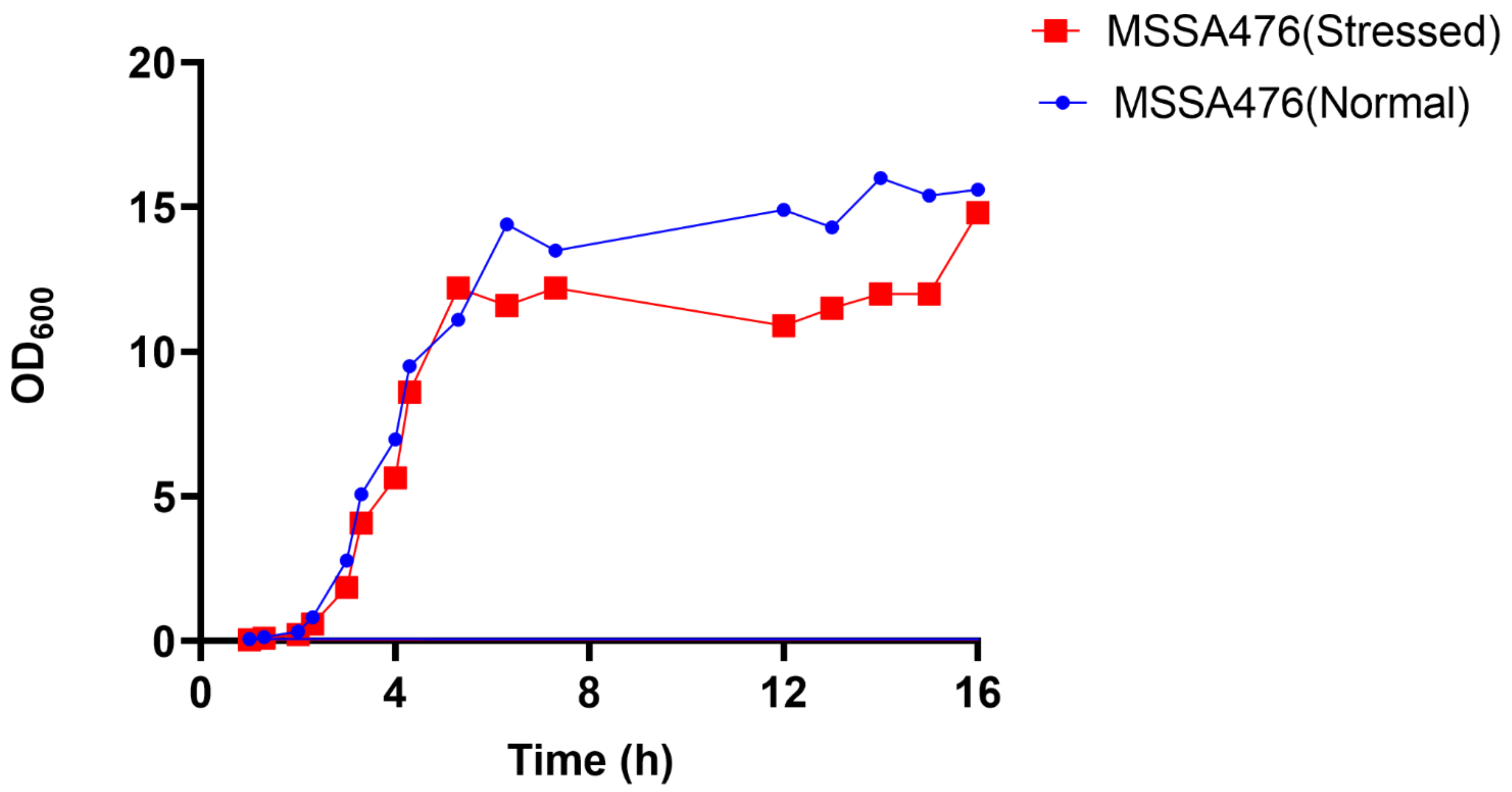
MSSA476(Stressed)
MSSA476(Normal)
20
15
10
5
0
OD600
0
4
8
12
16
Time (h)

Figure S2

A

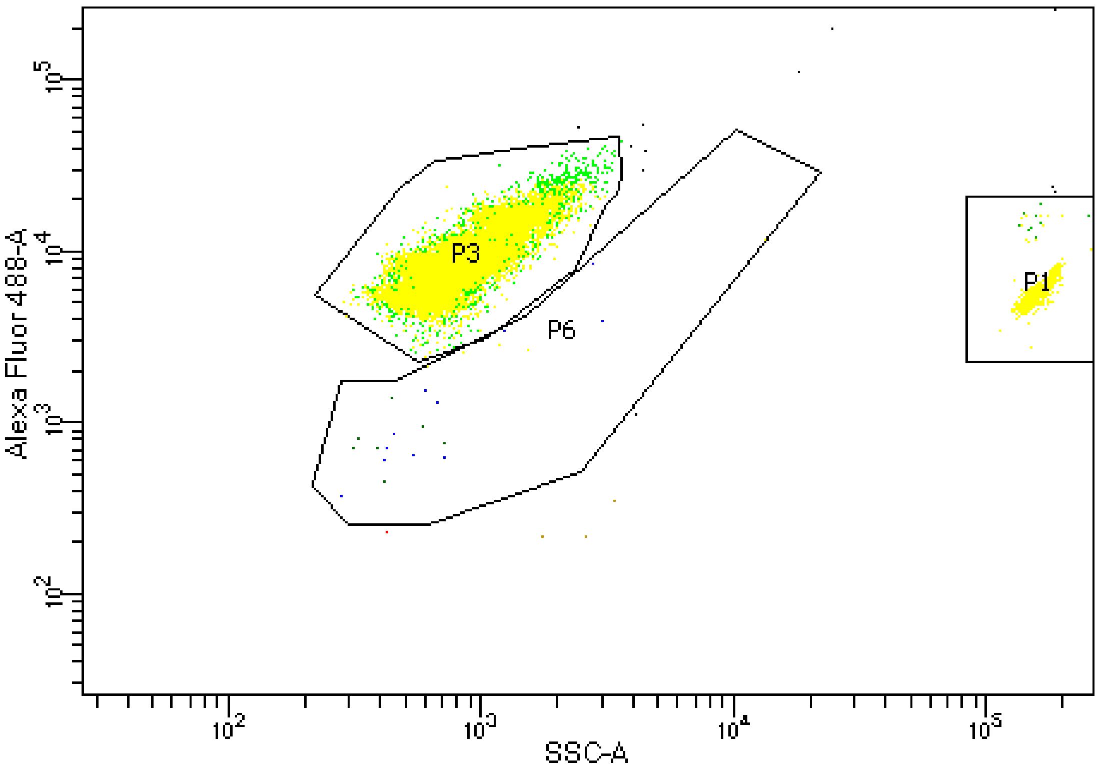

S. aureus grown under normal condition

B

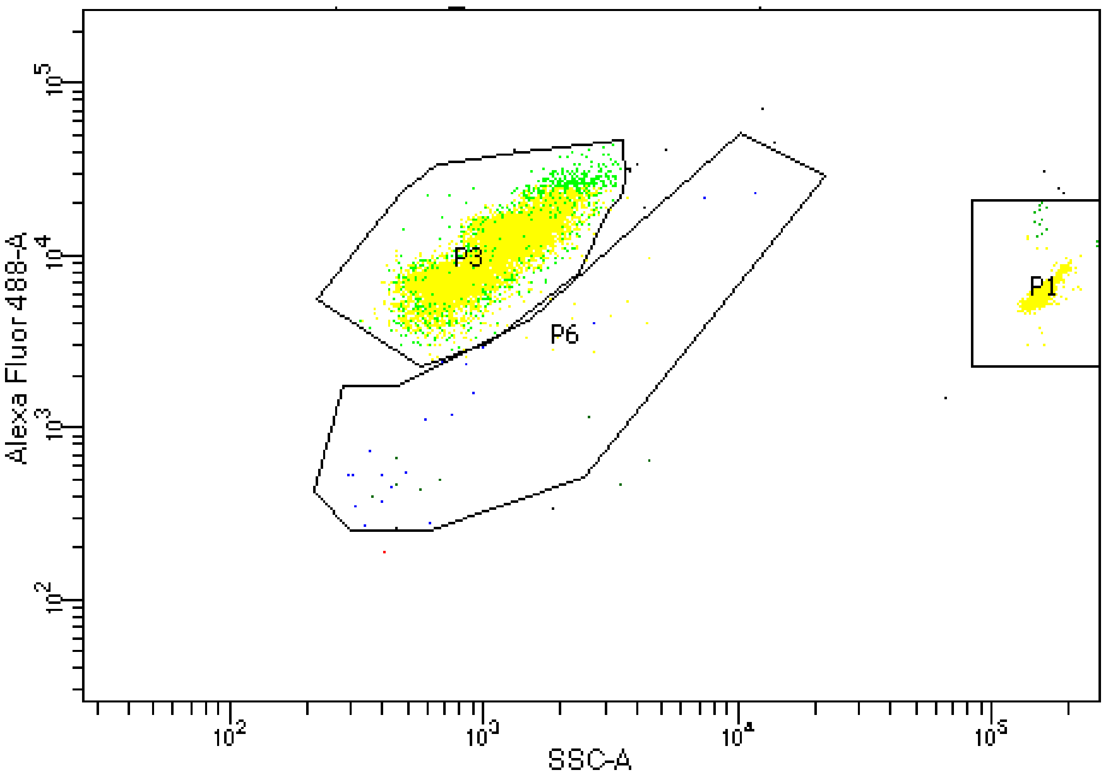

S. aureus grown under stressed condition

Figure S3

A

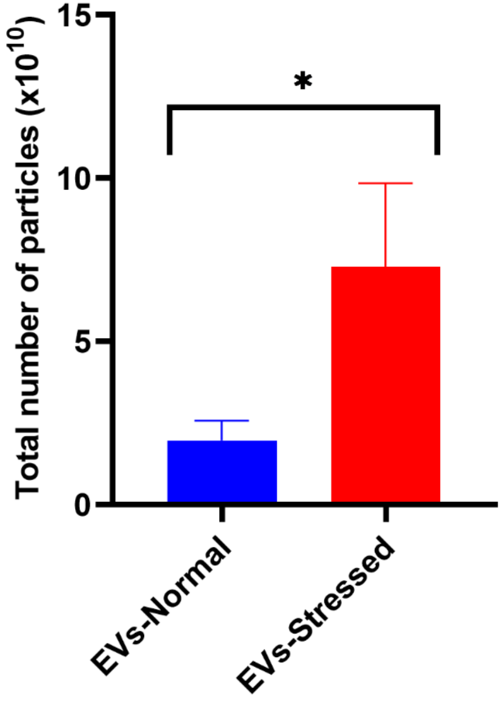
*
Total number of particles (x10^10)
15
10
5
0
EVs-Normal
EVs-Stressed

B

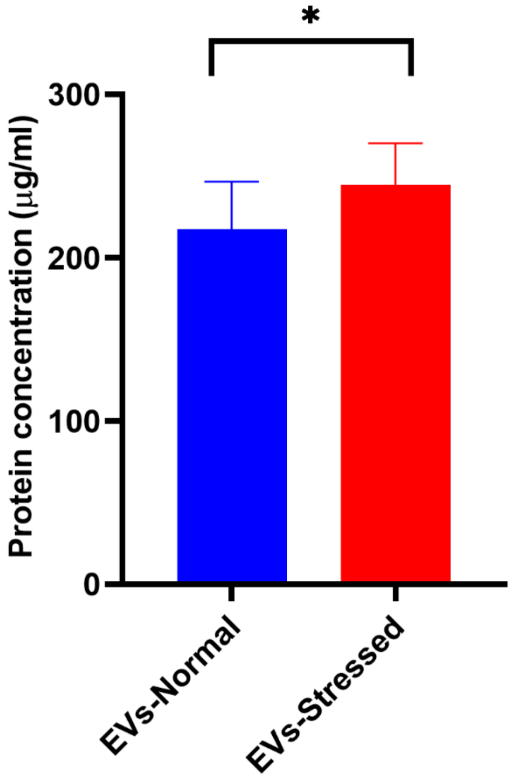
*
Protein concentration (μg/ml)
300
200
100
0
EVs-Normal
EVs-Stressed

Figure S4

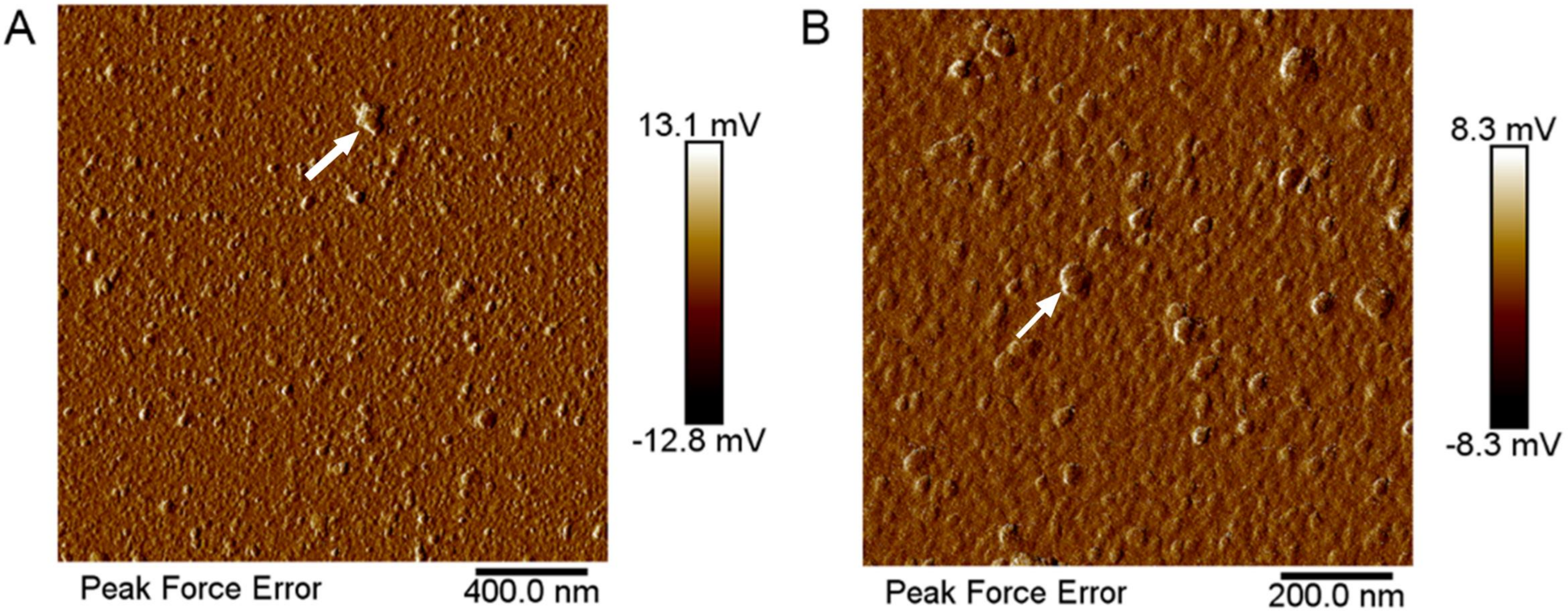
A
13.1 mV
-12.8 mV
Peak Force Error
400.0 nm
B
8.3 mV
-8.3 mV
Peak Force Error
200.0 nm

Figure S5

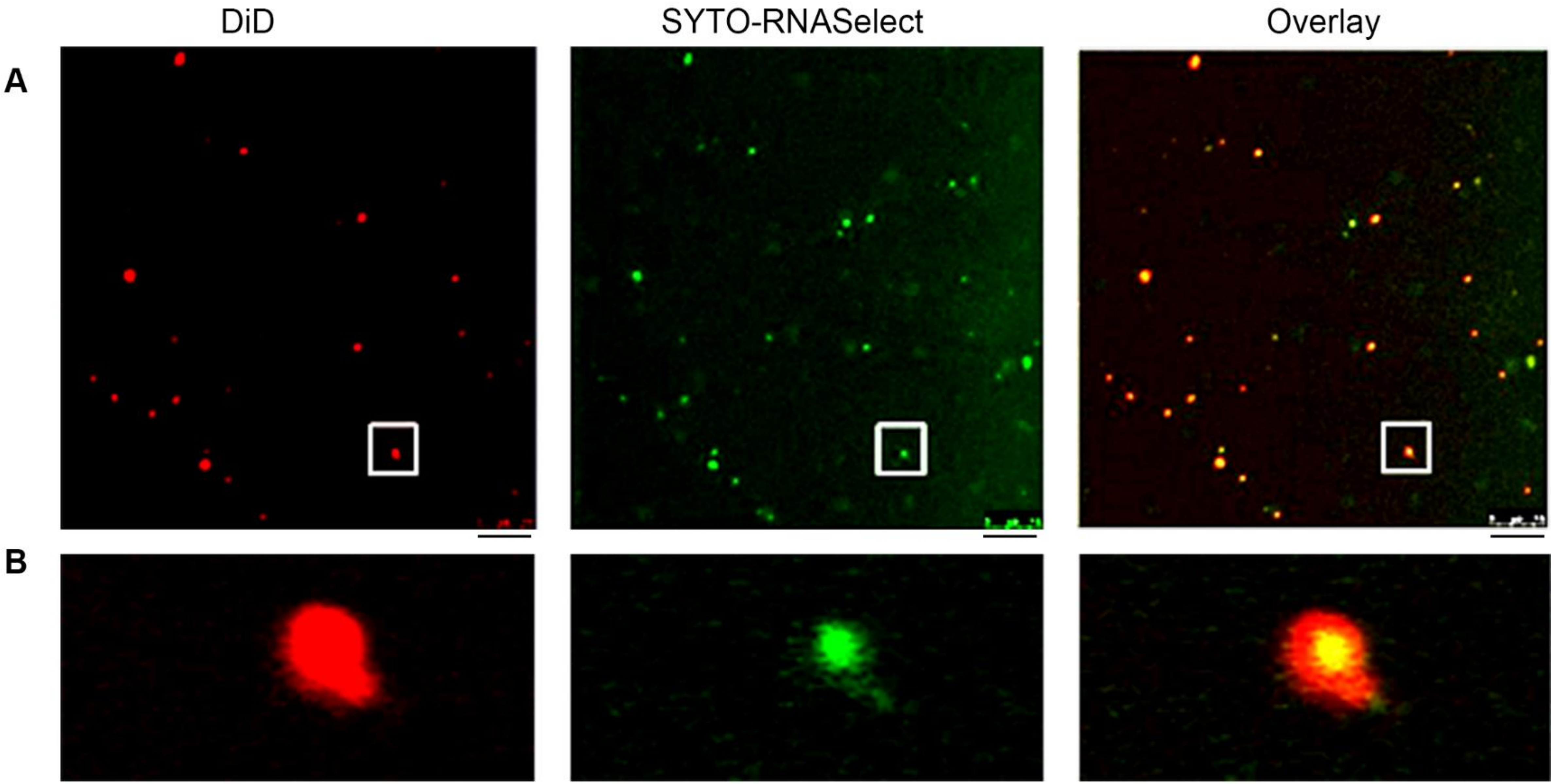

Figure S6

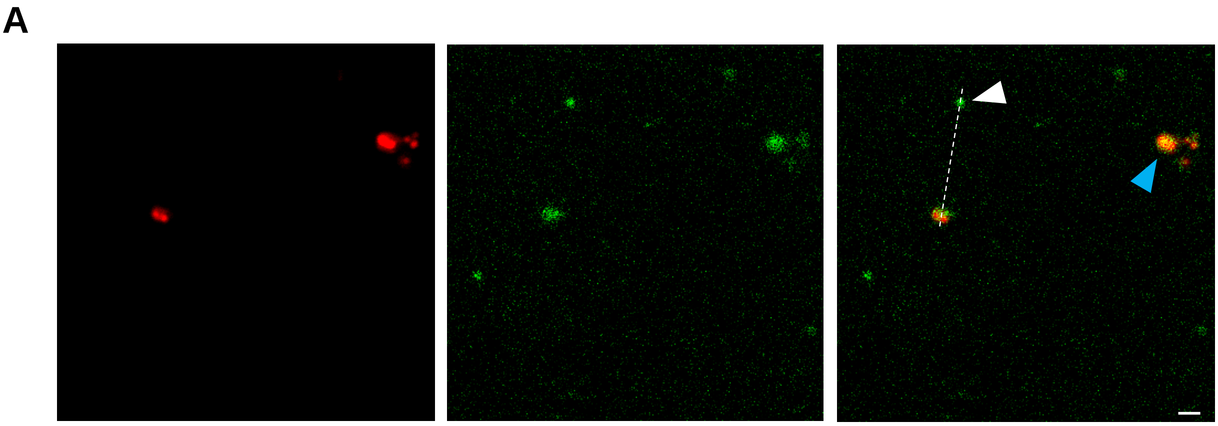
A

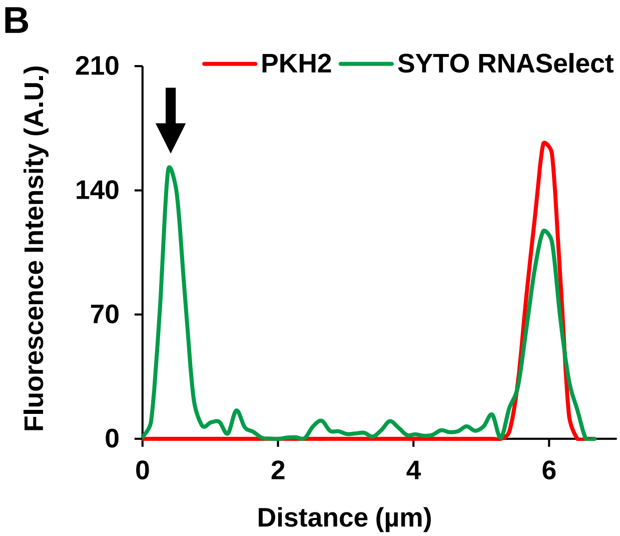
B
210
140
70
0
Fluorescence Intensity (A.U.)
PKH2
SYTO RNASelect
0
2
4
6
Distance (μm)

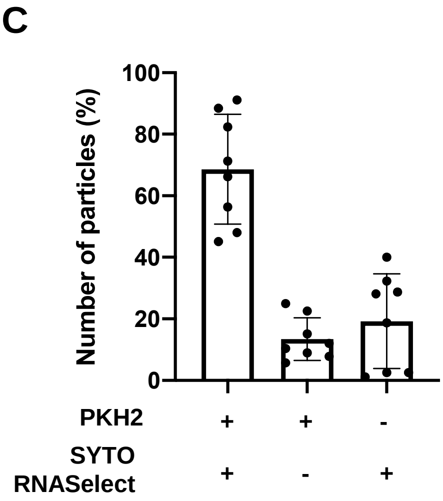
C
100
80
60
40
20
0
Number of particles (%)
PKH2
+
+
-
SYTO RNASelect
+
-
+

Figure S7

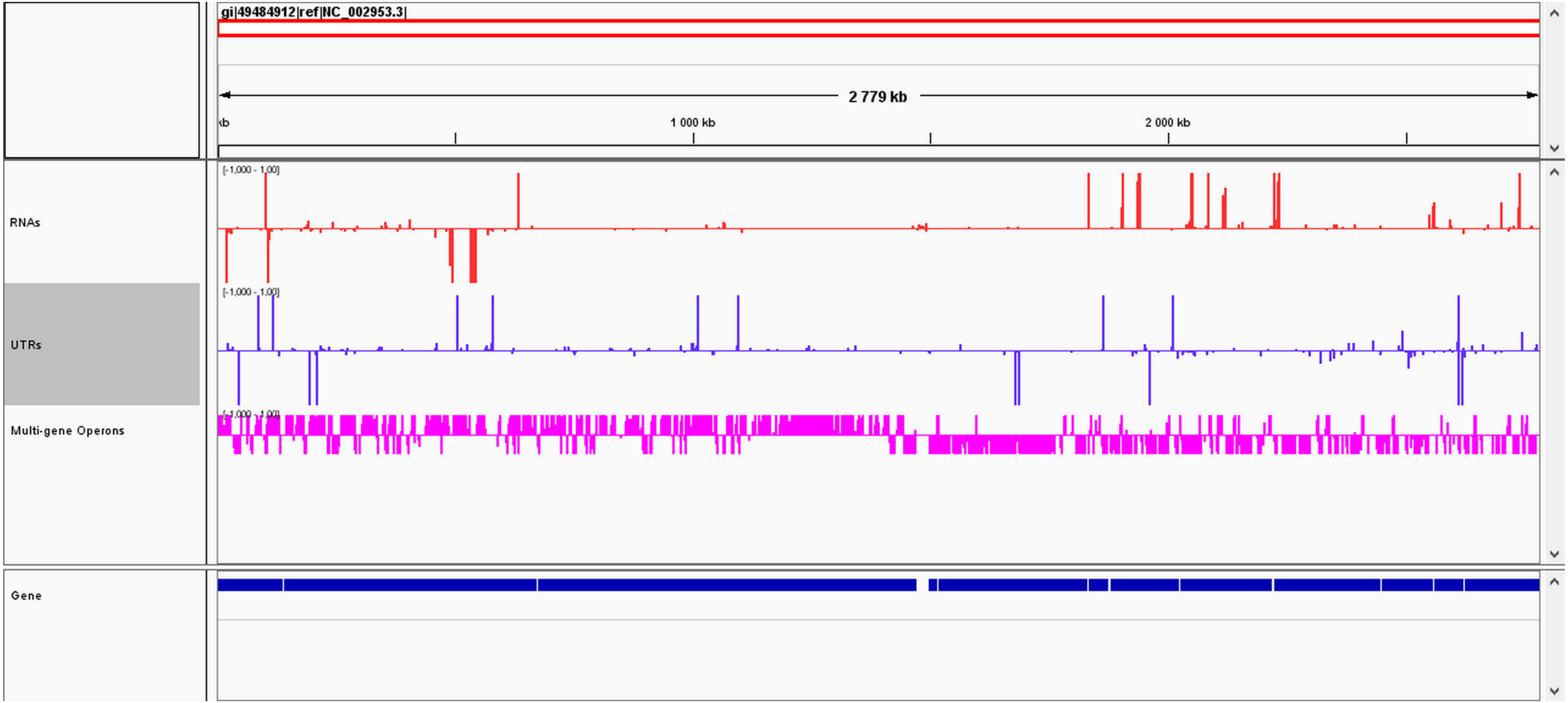

gi|49484912|ref|NC_002953.3|
2 779 kb
1 000 kb
2 000 kb
RNAs
UTRs
Multi-gene Operons
Gene

Figure S8

A

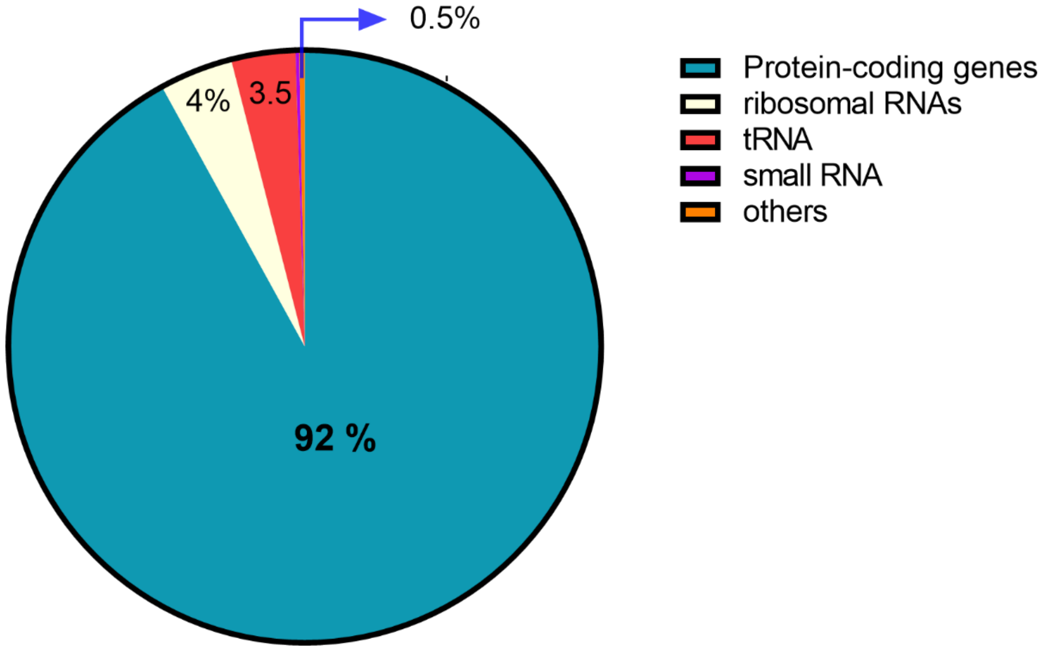

% Read mapped to chromosome

B

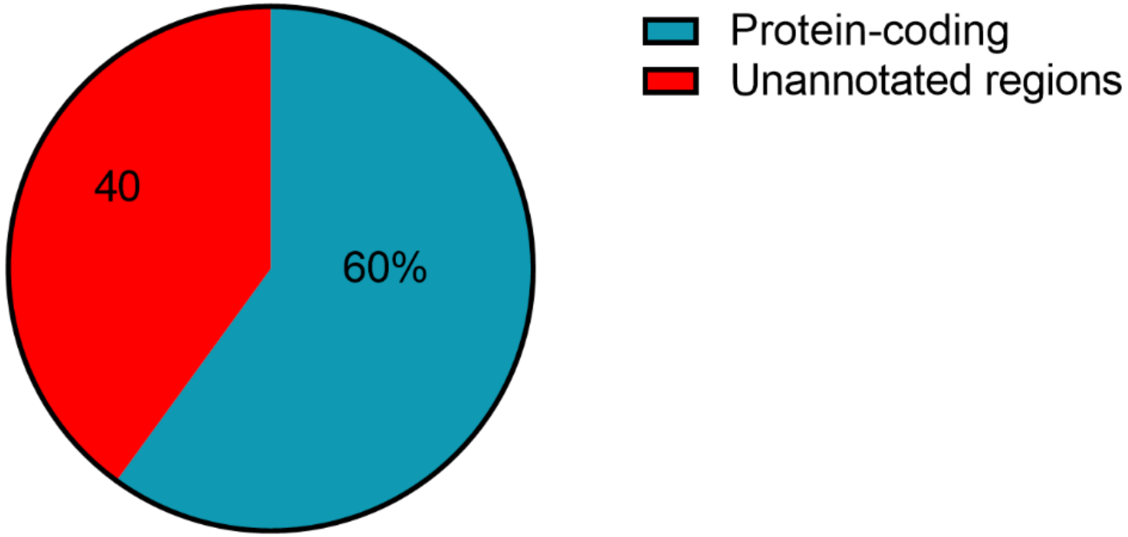

% Read mapped to plasmid

Figure S9

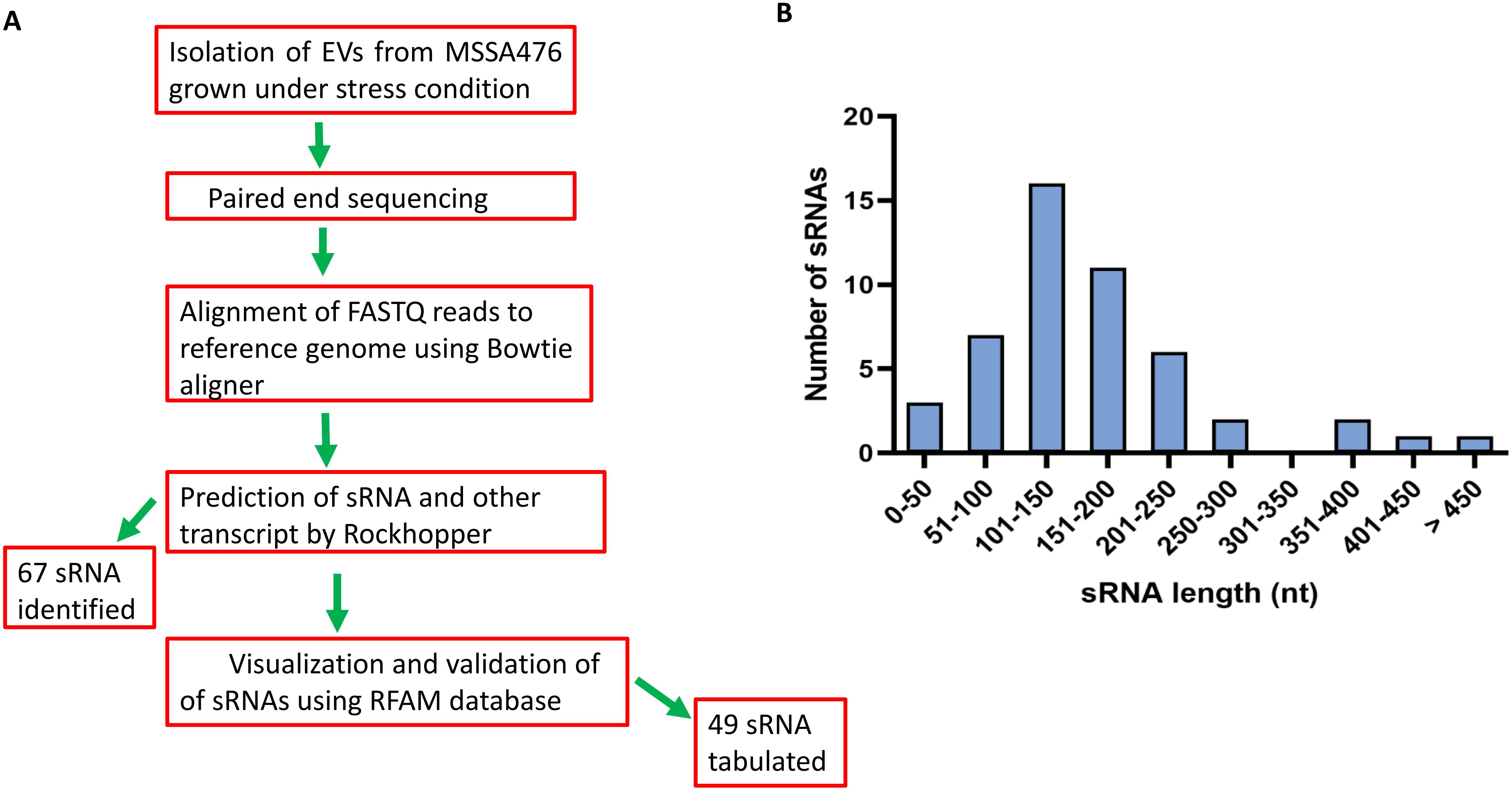

A
Isolation of EVs from MSSA476 grown under stress condition
Paired end sequencing
Alignment of FASTQ reads to reference genome using Bowtie aligner
Prediction of sRNA and other transcript by Rockhopper
67 sRNA identified
Visualization and validation of of sRNAs using RFAM database
49 sRNA tabulated
B
Number of sRNAs
0
5
10
15
20
0-50
51-100
101-150
151-200
201-250
250-300
301-350
351-400
401-450
> 450
sRNA length (nt)

Figure S10

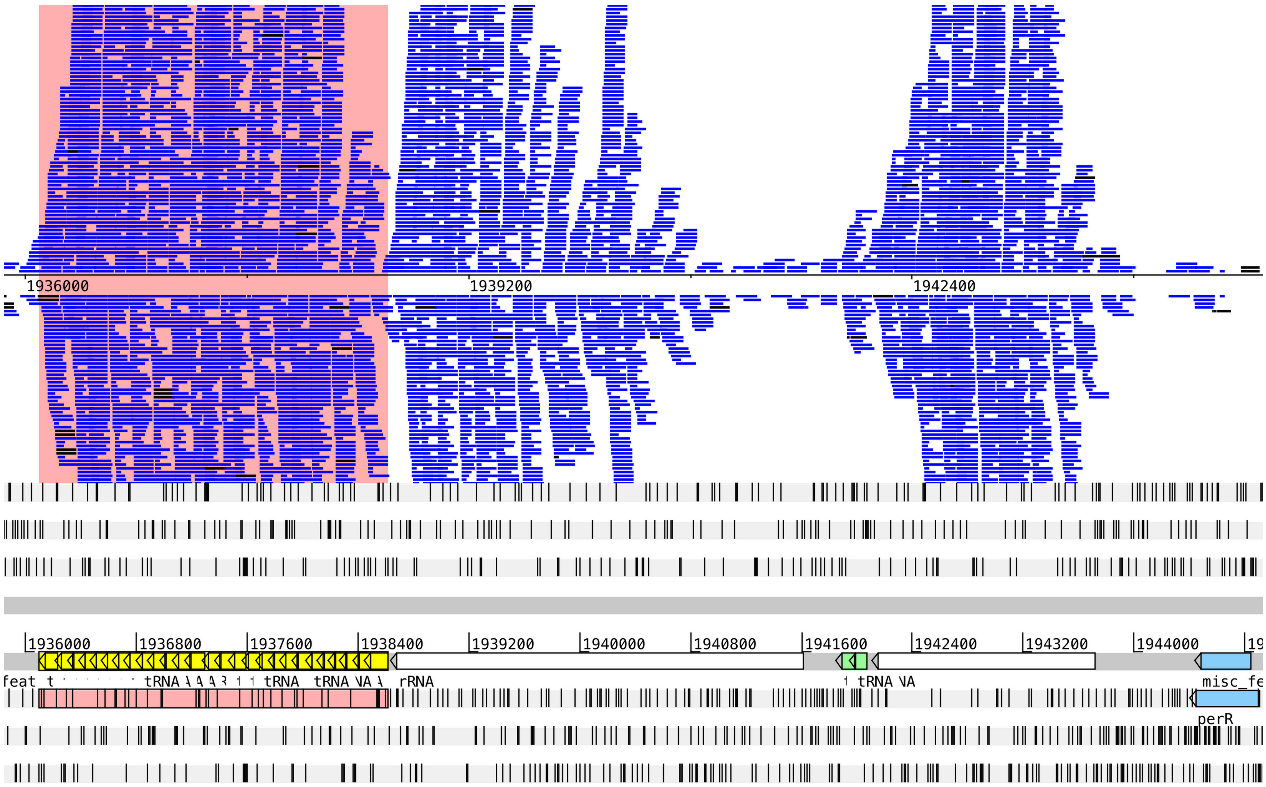
1936000
1939200
1942400
1936000
1936800
1937600
1938400
1939200
1940000
1940800
1941600
1942400
1943200
1944000
tRNA
tRNA
tRNA
rRNA
tRNA
misc_fe
perR

Figure S11

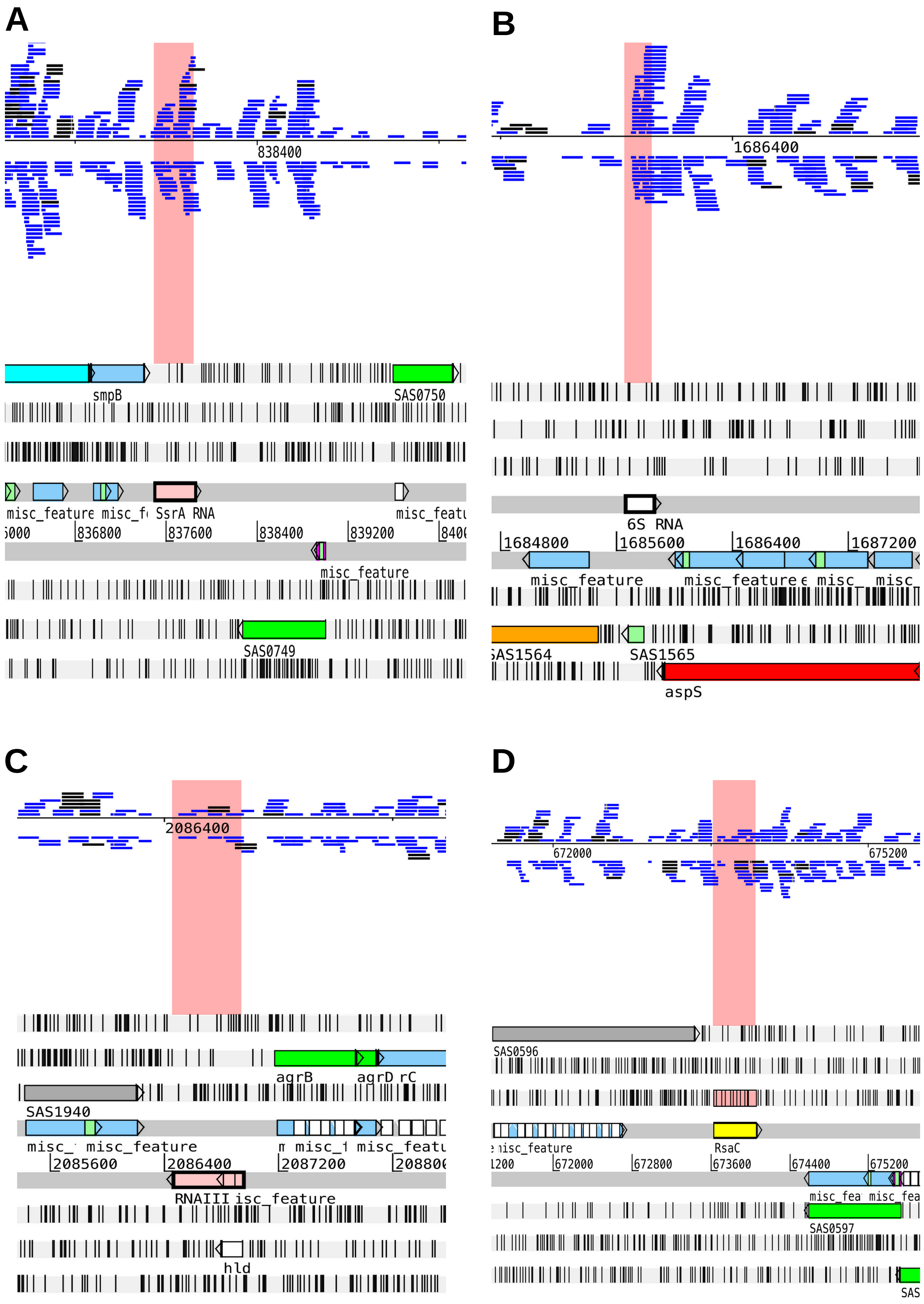
A
838400
smpB
SAS0750
misc_feature
SsrA RNA
836800
837600
838400
839200
SAS0749
B
1686400
6S RNA
1684800
1685600
1686400
1687200
misc_feature
SAS1564
SAS1565
aspS
C
2086400
agrB
SAS1940
2085600
2086400
2087200
RNAIII
hld
D
672000
675200
SAS0596
RsaC
672800
673600
674400
SAS0597

Figure S12

*ssr*A

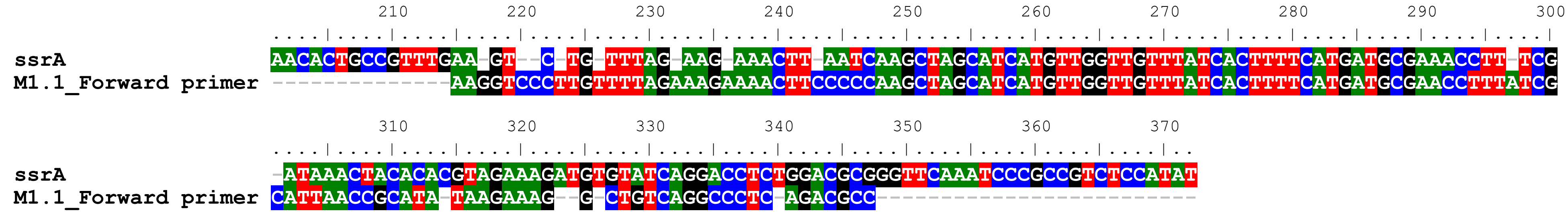

*rsa*C

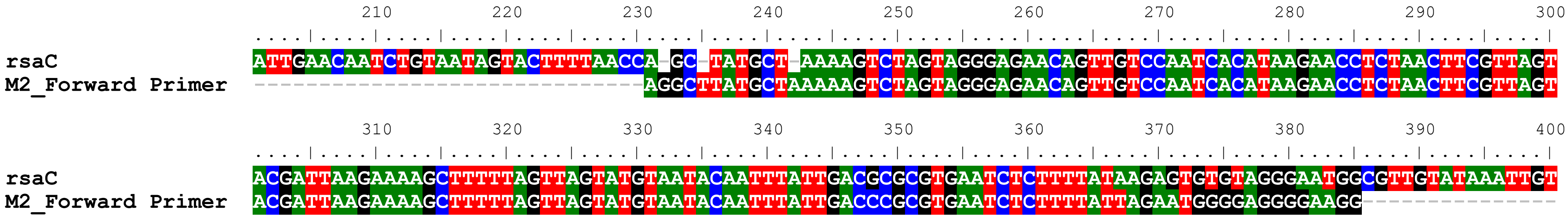

RNAIII

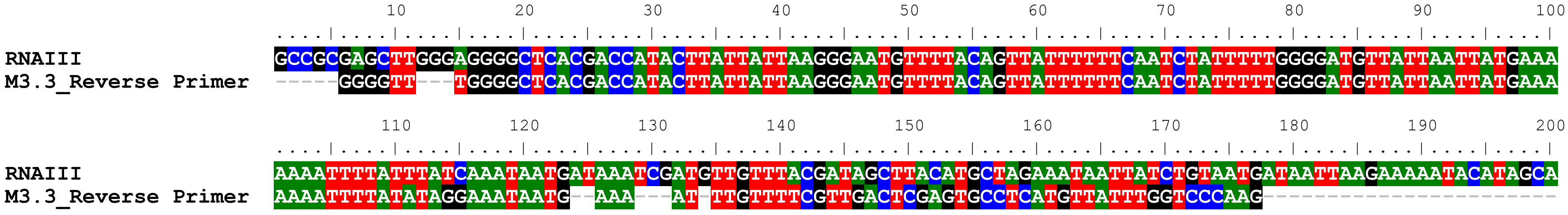

Supplement: Supplementary Figure 1 — Growth curves of S. aureus grown under BHI (normal condition) and iron-depleted BHI media with subinhibitory concentration of vancomycin (stressed condition). [file Data_Sheet_1.PDF]
